# Hot exciton dissociation in graphene nanoribbons

**DOI:** 10.1038/s41467-026-74158-x

**Published:** 2026-06-12

**Authors:** Guanzhao Wen, Fugui Xu, Alexander Tries, Wenhao Zheng, Lucia Di Virgilio, Shuai Fu, Xinyu Chen, Lin Yang, Zijie Xiao, Mathias Kläui, Silvio Osella, Ji Ma, Xu Wang, Xinliang Feng, Yiyong Mai, Mischa Bonn, Hai I. Wang

**Affiliations:** 1 Max Planck Institute for Polymer Research, Mainz, Germany; 2 State Key Laboratory of Synergistic Chem-Bio Synthesis, School of Chemistry and Chemical Engineering, Shanghai Key Laboratory of Electrical Insulation and Thermal Ageing, Shanghai Jiao Tong University, Shanghai, China; 3 Institute of Physics, Johannes Gutenberg-University Mainz, Mainz, Germany; 4 College of Polymer Science and Engineering, State Key Laboratory of Advanced Polymer Materials, Sichuan University, Chengdu, China; 5 Max Planck Institute of Microstructure Physics, Halle, Germany; 6 Materials and Processes Simulation Lab, Centre of New Technologies, University of Warsaw, Warsaw, Poland; 7 Beijing National Laboratory for Molecular Sciences, CAS Key Laboratory of Organic Solids, Institute of Chemistry, Chinese Academy of Sciences, Beijing, China; 8 Centre for Advancing Electronics Dresden & Faculty of Chemistry and Food Chemistry, Technische Universität Dresden, Dresden, Germany; 9 Nanophotonics, Debye Institute for Nanomaterials Science, Utrecht University, Utrecht, The Netherlands; 10 Present Address: Bundesdruckerei GmbH, Berlin, Germany

**Keywords:** Electronic properties and materials, Two-dimensional materials, Electronic properties and materials

## Abstract

Exciton dissociation in semiconducting nanostructures is crucial for optoelectronic applications, especially when free-carrier generation is required. Despite considerable research, the question of whether and how such generation occurs in strongly excitonic systems remains elusive. Here, we use one-dimensional precision graphene nanoribbons (GNRs) as a model system to investigate exciton dissociation. We systematically explore the interplay between ribbon length (*l*), excitation energy, and band dispersion in various precision GNRs. Ultrafast Terahertz conductivity measurements reveal that hot exciton dissociation dominates carrier generation, with ribbon length significantly influencing free carrier lifetimes. We identify a critical Bjerrum length (*R*_B_) of approximately 20 nm that determines whether photoexcited hot carriers in GNRs can dissociate before forming tightly bound excitons. For shorter ribbons (*l* < 2*R*_B_), rapid ~ps exciton formation prevails. Furthermore, the charge-carrier band dispersion in GNRs plays a critical role in determining dissociation efficiency. Long GNRs with strongly dispersed bands, and consequently low effective carrier masses, exhibit higher mobilities that promote efficient hot-exciton dissociation. These results advance fundamental understanding of dimensionality, energetics, and electronic structure in excitonic materials, providing design principles for optoelectronic devices based on excitonic materials.

## Abstract (web summary)

Using terahertz spectroscopy, the authors show that hot exciton dissociation dominates carrier generation in graphene nanoribbons, with excitation excess energy, ribbon length, and band dispersion jointly governing generation yield and lifetime.

## Introduction

Efficient conversion of excitons into free charge carriers is essential for many optoelectronic devices, such as solar cells and photodetectors^1–5^. These technologies are increasingly reliant on low-dimensional materials^6–8^, where reduced dimensionality suppresses dielectric screening, particularly in materials with inherently low permittivity, such as organic or hybrid systems. This reduced screening results in enhanced Coulomb interactions and, consequently, substantially higher exciton binding energies (*E*_B_) than in bulk materials or traditional semiconductors, which poses a significant barrier to generating mobile charges. Hence, understanding and overcoming the challenges of exciton dissociation in these low-dimensional systems is central to advancing the design and performance of next-generation excitonic devices.

In low-dimensional organic-material systems, *E*_B_ typically ranges from 0.1 to 1 eV, depending on the degree of confinement and the material system^9–12^. A large *E*_B_ is advantageous for sustaining tightly bound excitons in light-emitting applications^13–15^, whereas it hinders photovoltaic and photodetection technologies, where efficient exciton dissociation into free carriers is essential. Exciton dissociation has therefore been identified as a key limiting step in device operation. Although some initial efforts have been devoted to understanding the exciton splitting process in various excitonic systems, including semiconducting polymers^16,17^, quantum dots^18–20^, and two-dimensional transition-metal dichalcogenides^21–24^, the underlying mechanisms that facilitate free charge generation have remained elusive. Moessner et al. proposed that the generation of free electrons and holes from excitons can be analogous to the dissociation of a free ions from ion pairs in an electrolyte^25^. By applying Onsager’s theory of field-assisted dissociation for ions, the authors described the enhanced free carrier escape probability under an external electric field, which lowers the Coulomb potential barrier between electron-hole pairs to facilitate their separation. Despite theoretical progress, little experimental evidence has been provided to support this scenario, and examples of experimentally tuning free carrier/exciton dynamics remain limited.

Graphene nanoribbons (GNRs) are narrow strips of graphene in which the lateral confinement opens an electronic bandgap that depends on ribbon width and edge geometry. Unlike most other low-dimensional materials, GNRs offer exceptional atomic-level structural precision, allowing for the systematic tuning of their electronic, magnetic, and optical properties by fine-tuning their atomic structural characteristics, including edge configurations, width, length, and atomic substitution^26–29^. Recent advances in bottom-up synthesis have delivered GNRs with controllable widths down to ~1 nm and tunable band gaps spanning 1–3 eV, greatly expanding the opportunities for physical property exploration and device integration. This strict confinement ensures that charge separation dynamics unfold exclusively along a single spatial axis, providing a direct, quantifiable link between exciton behavior and ribbon length. Recent spectroscopic studies have revealed extraordinarily strong exciton effects with a binding energy *E*_B_ on the order of 1 eV^11,30,31^ (a direct consequence of suppressed screening and pronounced quantum confinement)^12,32^, establishing GNRs as an ideal model to probe the interplay between dimension, structure, electronic structure, and exciton dynamics in low-dimensional systems.

In addition to tuning the edge structure and width of GNRs, engineering ribbon length (*l*) can provide significant control over their electronic and optical properties^33–35^. Several previous calculations have revealed how the bandgap evolves as the length of GNRs is gradually extended, starting from the monomer^36–39^. While a strong confinement effect in the length direction has been demonstrated for short ribbons, the bandgap energy of GNRs converges to its bulk value for lengths beyond a few nanometers^37,38^. For instance, combining experimental and theoretical studies, Talirz et al. reported that when the ribbon length of *N*_A_ = 7 armchair GNRs reaches ~8 nm, the bandgap converges to within ~50 meV of the infinite-length value, indicating a saturation in the dependence of bandgap on length^38^. Consequently, bandgap tunability through length engineering is limited to relatively short ribbons. In other words, for sufficiently long ribbons (e.g., *l* > 10 nm) with great potential for electronic applications, further extending the length is not expected to have a significant impact on the electronic properties of GNRs. However, whether and how the ribbon length influences the non-equilibrium electronic properties remains unexplored.

In this work, we investigate a length-dependent effect on non-equilibrium hot carrier transport and dissociation in GNRs via ultrafast Terahertz (THz) conductivity measurements following photo-injection of charge carriers. The THz conductivity provides access to the photoinduced complex conductivity of the GNRs, with distinct signatures from free carriers — exhibiting finite real and imaginary components—and excitons, characterized by a near-zero real and finite imaginary component in the few-THz range. While direct optical excitation above the electronic band gap can inject free carriers into GNRs, they typically recombine or undergo rapid localization on the picosecond (ps) timescale^11,40,41^, restricting the time window for charge extraction in practical devices. Overcoming this bottleneck and establishing design principles for generating long-lived, mobile carriers is thus central to advancing GNR-based photonic and electronic technologies. This motivates a systematic investigation of how intrinsic factors, such as ribbon length, the energetics of hot carriers, and electronic structure, govern the efficiency and lifetime of exciton dissociation and free charge generation in GNRs.

**Fig. 1:**
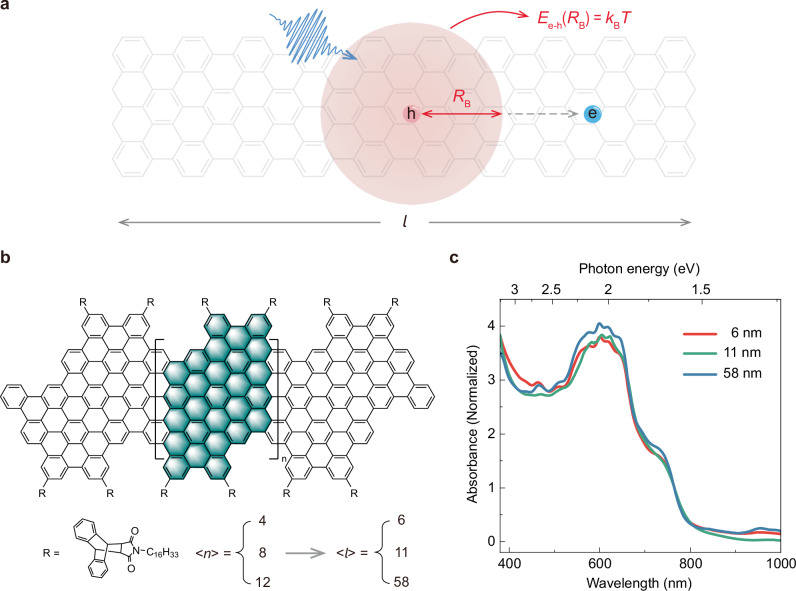
Molecular design of length-defined AHM-GNRs. **a** Illustration of hot exciton dissociation and free carrier generation in AHM-GNRs. Here, *l* represents the length of GNRs. *R*_B_ defines the electron-hole distance at which the Coulomb potential energy (*E*_e-h_) is equal to the thermal excitation energy *k*_B_*T*. **b** The molecular structure of the three AHM-GNRs used in this work. <*n>* represents the average number of repeating units. **c** Ultraviolet-visible-near-infrared absorption spectra of AHM-GNR samples. The spectra are normalized to the first exciton transition at ~ 765 nm (or equivalently ~1.62 eV).

We discover a critical length scale (*R*_B_, the exciton Bjerrum length, as shown in Fig. 1a and subsequent discussion) in GNRs that determines whether hot exciton dissociation (HED) can occur effectively to generate spatially separated free electrons and holes. *R*_B_ (~ 20 nm) is found to extend far beyond the conventional quantum-confinement length scale (~1 nm) in GNRs and is effectively determined by the spatial extent of the weakly screened electron-hole Coulomb potential. For short ribbons with *l* < 2*R*_B_, the charge separation by the HED effect is inherently restricted by the ribbon length. This limitation results in the ultrafast formation of bound excitons on the ps time scale with near-unity quantum yields. On the other hand, for GNRs with *l* > 2*R*_B_, the kinetic energy of hot carriers can effectively facilitate charge separation to the extent that the electrons no longer experience significant electrostatic interaction with the holes. We further reveal the decisive role of GNR band dispersion and consequently their carrier effective mass in determining HED efficiency: low-effective-mass GNRs exhibit enhanced mobility, which favors the kinetic competition between HED and thermalization, thereby increasing HED efficiency. Our results can be qualitatively rationalized using Onsager’s theory of field-assisted dissociation of electrostatically sticky excitons^25^. These findings also provide critical insights into the role of length on the efficient, long-lived free carrier generation in GNRs, which is crucial for their applications in optoelectronics.

## Results

### Quantifying length-dependent carrier transport effect

In this work, the studied GNRs have a fixed width of 1.7 nm and average lengths of 6, 11, and 58 nm controlled by the number of repeating units of 4, 8, and 42, respectively. The corresponding molecular weights and polydispersities of the polymer precursors are summarized in Supplementary Table 1. A sketch of the investigated GNRs is shown in the inset of Fig. 1b. The GNRs functionalized with Diels-Alder cycloadducts of anthracenyl units and *N*-n-hexadecylmaleimide (AHM) (see synthetic details in ref. ^42^) are used to promote single-ribbon dispersion of GNRs in organic solvents (toluene in this study). The three available ribbon lengths enable us to investigate length-dependent charge-carrier dynamics in AHM-GNRs spanning aspect ratios by an order of magnitude (from ~ 3 to 35). The uniformity of AHM-GNRs was demonstrated by atomic force microscopy and Raman spectroscopy in our previous study^42^. Fig. 1c shows the measured absorbance spectra for all samples. In contrast to the ~2.3% wavelength-independent absorption of monolayer suspended graphene in the visible-infrared range, the AHM-GNRs exhibit a semiconducting response: increasing the ribbon average length from ~6 to ~58 nm yields almost the same optical bandgap of ~1.62 eV. In line with previous reports, our results indicate that the length scale plays a negligible role in determining the electronic and optical properties of AHM-GNRs with *l* > 6 nm^33,35^. Instead, the properties of the AHM-GNRs are dominated by their nature and the geometrical confinement along the narrow width.

To quantify the length-dependent free carrier and exciton dynamics, we conduct optical pump-THz probe (OPTP) spectroscopy. We optically inject charge carriers into GNRs by excitation with tunable photon energies (from 1.62 eV to 3.1 eV). The complex (real and imaginary) conductivity of photogenerated carriers (Δ*σ*) is further probed by a single-cycle THz field (*E*, with a duration of ~ 1 ps), following the correlation between the real and imaginary parts of *σ* and the photoinduced relative absorption and temporal shift, respectively, of the THz field (−Δ*E*/*E*). We disentangle free carriers from excitons by their distinct responses at THz frequencies^11,43,44^. In principle, the angular frequency (*ω*) dependent intra-excitonic absorption (or equivalent conductivity), e.g., the transition between 1 *s* and 2*p* exciton states of materials, can be well-described by the Lorentzian resonance:

1 $${\sigma }_{{\mbox{EX}}}\left(\omega \right)=\frac{{\omega }_{{\mbox{p}}}^{2}{\varepsilon }_{0}\tau }{1-i\omega \tau+i\tau {\omega }_{0}^{2}/\omega }$$

where *τ*, *ω*_p_, *ω*_0_, and *ε*_0_ are the relaxation time, plasma frequency, resonant frequency, and vacuum permittivity, respectively. Given the substantial intra-excitonic splitting (hundreds of meV) and the narrow bandwidth of our spectrometer (up to ~ 2.5 THz, ~ 10 meV)^11,41^, we can restrict the analysis to the low-frequency limit (*ω* ≪ *ω*_0_). In addition, the 1*s*−2*p* resonance in GNRs exhibits a high-quality factor (*ω*_0_*τ* ≫ 1), such that the term, $i\tau {\omega }_{0}^{2}/\omega$, dominates the denominator of Eq. 1. Under these conditions, we can rewrite Eq. 1 as:

2 $${\sigma }_{{\mbox{EX}}}\left(\omega \right)\approx -i\omega {\varepsilon }_{0}({\omega }_{{\mbox{p}}}^{2}/{\omega }_{0}^{2})$$

This result gives a nearly negligible real part of the photoconductivity and a negative finite imaginary part whose amplitude increases linearly with frequency for excitons in GNRs. It has been shown that the slope is proportional to the excitonic polarizability (*α*_0_), given by ${\alpha }_{0}={\omega }_{{\mbox{p}}}^{2}/{\omega }_{0}^{2}$^44,45^. On the other hand, free carriers have a considerable contribution to the real part of the photoconductivity, which can be well described by the modified Drude model (so-called Drude-Smith model) following^46,47^:

3 $${\sigma }_{{\mbox{F}}}\left(\omega \right)=\frac{{\omega }_{{\mbox{p}}}^{2}{\varepsilon }_{0}{\tau }_{{\mbox{DS}}}}{1-i\omega {\tau }_{{\mbox{DS}}}}\left(1+\frac{c}{1-i\omega {\tau }_{{\mbox{DS}}}}\right)$$

where *τ*_DS_ represents the charge scattering time, and the parameter *c* represents the probability of backscattering at defects and/or edge boundaries, with values spanning from 0 (for isotropic scattering) to −1 (for preferential scattering). In essence, this model accounts for the preferential backscattering effect, e.g., due to geometrical confinement in one-dimensional structures, which has previously been applied to carbon nanostructures such as carbon nanotubes and GNRs^41,48–51^. The distinct spectroscopic signatures of excitons and free carriers, particularly in the real and imaginary parts of the photoconductivity, make OPTP spectroscopy ideally suited as a sensitive and convenient probe for studying the nature and dynamics of photogenerated charge species in GNRs.

**Fig. 2:**
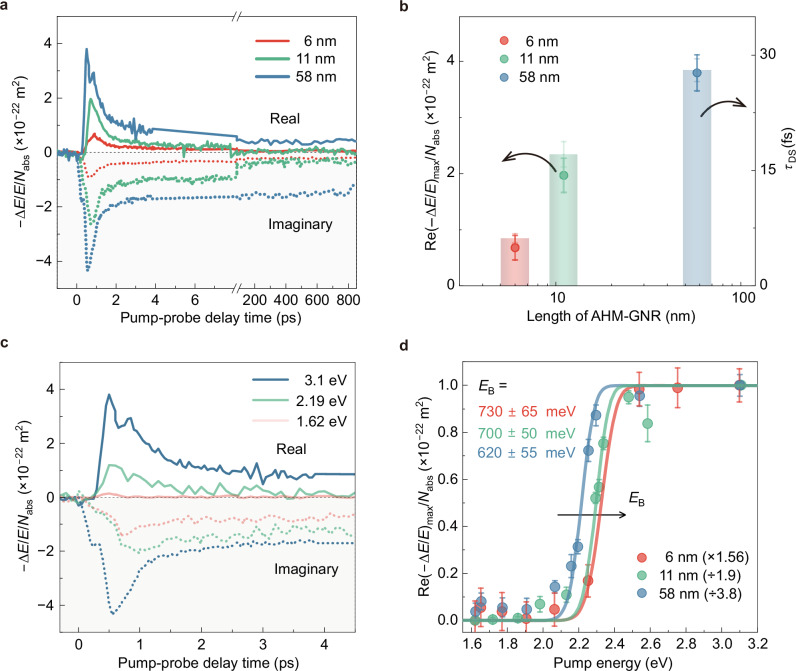
Length-dependent free-carrier generation in AHM-GNRs. **a** Time-resolved THz photoconductivity of AHM-GNR with different lengths normalized to absorbed photon density (*N*_abs_) after 3.1 eV excitation. **b** Left ordinate axis (round data points), the maximum value of the real part of −Δ*E*/*E*/*N*_abs_ corresponding to **a**. The error bars represent the standard error of the maximum value for multiple measurements; Right ordinate axis (vertical bars), the charge scattering time inferred from the Drude-Smith model. **c** *hv*-dependent THz photoconductivity of AHM-GNR with a length of 58 nm. **d** The maximum value of the real part of −Δ*E*/*E*/*N*_abs_ (scaled by the indicated factors for visual comparison) after *hv*-dependent excitations. The error bars represent the standard error of the maximum value for multiple measurements. The solid lines are the fitting curves based on the model described in ref. ^11^. Data for the 11 nm GNR in panels **a** and **d** are adapted from ref. ^11^, licensed under CC-BY.

Using OPTP spectroscopy, we obtained a comprehensive overview of the photoinduced charge-carrier and exciton dynamics in AHM-GNRs of different lengths (see Fig. 2). Figure 2a displays the time-resolved THz photoconductivity for ribbons with average lengths of ~6, ~11, and ~58 nm, each normalized to the absorbed photon density (*N*_abs_, see Supplementary Note 1 for details) after excitation with a pump-photon energy of 3.1 eV. The results reveal that the peak of real photoconductivity increases with GNR length, indicating that longer ribbons support higher free carrier mobility and longer-lived free carriers. We note that within the range of pump fluences used in our studies, for a given pump photon energy *hν*, all photoconductivity dynamics scale with the absorbed photon density. Specifically, the peak photoconductivity values scale linearly with *N*_abs_ (see Supplementary Fig. 1), while the normalized dynamics overlap with one another (see Supplementary Fig. 2). Figure 2b presents a quantitative summary: the left axis shows the maximum real part of the photoconductivity (normalized by *N*_abs_) for each GNR, while the right axis plots the charge carrier scattering time, *τ*_DS_, extracted from model fits to the photoconductivity spectra measured at ~0.5 ps after the maximum photoconductivity (where the free carrier response dominates the THz photoconductivity; see Supplementary Note 2, Supplementary Fig. 3, and Supplementary Table 2). In short, at a given pump-probe delay, we record the entire waveform of the transmitted THz field with (*E*_P_(*t*)) and without (*E*_0_(*t*)) optical excitation. Following the Fourier transformation, the photoinduced change of the THz field in the frequency domain ∆*E*(*ω*) *= E*_P_(*ω*) − *E*_0_(*ω*) can be obtained and is directly proportional to the photoconductivity at the THz frequency ∆*σ*(*ω*)^52^. Fits of ∆*σ*(*ω*) to Eq. (3) provide access to the scattering time *τ*_DS_. Both the maximum real photoconductivity and *τ*_DS_ consistently rise with increasing ribbon length, highlighting reduced end-scattering as the origin of the enhanced mobility in longer GNRs. This conclusion agrees with our recent study on length-dependent charge transport in narrow molecular wires, wherein we demonstrated that charge transport in short GNRs (e.g., with sub-10-nm lengths) is limited by scattering at the ribbon ends. In contrast, momentum scattering from defects, local structural deformations, and phonons within the ribbons dominates conduction in longer GNRs^34,51^. In this work, we applied the Drude-Smith model to capture the complex interplay of scattering mechanisms. Because the GNRs form a disordered ensemble in solution, orientational averaging of the one-dimensional (1D) carrier motion inherently yields a geometric backscattering footprint of *c* $\approx$ − 0.79^41^. The extracted *c* values (various −0.91 to −0.96) exceed this purely geometric limit, reflecting additional physical backscattering that suppresses long-range transport. This excess backscattering arises from conformational bends or defects in extended ribbons, whereas finite-length boundary (end) scattering is expected to become more important in shorter ribbons^41^. Accordingly, the Drude-Smith model serves as a suitable phenomenological tool to account for the suppressed *dc* conductivity originating from both geometric projection and intra-ribbon scatterins.

### Quantifying length-dependent *E*_B_ in GNRs

To further investigate how excess photon energy affects carrier generation and transport in GNRs, we examine the time-resolved photoconductivity of AHM-GNRs as a function of excitation photon energy (*hν*). Figure 2c, Supplementary Fig. 1, and Supplementary Fig. 2 illustrate the *hν*-dependent THz photoconductivity for AHM-GNR with 58 nm. In good agreement with our previous report^11^, when excited at the optical bandgap (*hν* = ~1.62 eV), the conductivity response is dominated by excitons with a negative imaginary component and a negligible real component. In contrast, higher photon energies (*hν* > 2.2 eV) create transient free carriers, which are visible as a finite real part in the photoconductivity spectrum. Clearly, free carriers are generated through the HED process, requiring sufficient excess energy in the nascent electron-hole pairs to overcome the Coulomb attraction. We then quantitatively evaluate the energetic threshold for this transition from excitonic (insulating) to free-carrier (conductive) behavior by tracking the maximum photoconductivity as a function of *hν* and ribbon length. Figure 2d summarizes these dependencies, revealing a distinct crossover once the *hν* surpasses a critical value above the bandgap. Taking into account the role of thermal excitation (*k*_B_*T*) and the energies of exciton states and the electronic band edges, we can quantitatively infer *E*_B_ of AHM-GNRs by fitting the free carrier generation efficiency under varied excitations (see details in Ref. ^11^). The fits, shown as dashed lines in Fig. 2d, yield *E*_B_ values of 730 ± 65, 700 ± 50, and 620 ± 55 meV for AHM-GNRs with lengths of 6, 11, and 58 nm, respectively. Such an inverse scaling between *E*_B_ and *l* can be qualitatively understood as a consequence of the increased delocalization of electron-hole wavefunctions and the reduced quantum confinement that occurs with increasing ribbon length^53^. Given the dense and nonhydrogenic excitonic spectrum inherent to GNRs, the optical transition to the continuum can be broadened by higher-lying excited states and resonances. Hence, the *E*_B_ values extracted here represent effective exciton binding energies rather than strict ground-state quantities. Moreover, we note that the *E*_B_ for the longest ribbons remains large, over 600 meV, demonstrating the general exciton-dominated optical properties of narrow, atomically precise GNRs. These results reveal the key influence of ribbon length and excitation energy on both the nature and dynamics of photogenerated charge species in GNRs.

**Fig. 3:**
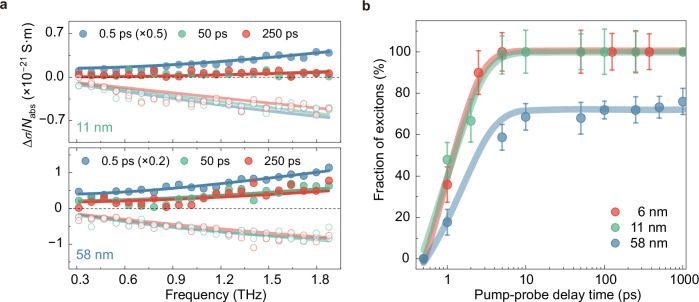
Length-dependent exciton reformation dynamics in AHM-GNRs. **a** Complex frequency-resolved THz photoconductivity of AHM-GNRs (with lengths of ~11 nm and ~58 nm) at ~0.5, ~50, and ~250 ps after the maximum THz photoconductivity following 3.1 eV excitation. The amplitudes of real (solid dots) and imaginary (open dots) parts of photoconductivity at ~0.5 ps are scaled by the indicated factors for visual comparison. The solid lines are fitted to the model described in the main text for photoconductivity cuts at 0.5, 50, and 250 ps, respectively. **b** The relative percentage of excitons as a function of the pump-probe delay time. The error bars are calculated via the standard error propagation from the uncertainties of *A*(*t*) and *B*(*t*). The solid lines represent fits to a single exponential rise function, *y*(*t*) = *A*[1 – exp(−*kt*)], where *A* is the saturation amplitude and *k* represents the characteristic rise rate. This functional form describes the temporal build-up of the exciton fraction, reflecting a single characteristic conversion process from free carriers to excitonic states.

To analyze how free carriers and excitons evolve over time in AHM-GNR samples, we investigate the frequency-resolved photoconductivity response after excitation at 3.1 eV (see Supplementary Note 2 for details)^41,48,54^. Fig. 3a compares the photoconductivity spectra of AHM-GNRs with lengths of 11 and 58 nm, revealing distinct long-lived charge states that form after excitation. For short GNRs, excitons dominate the response: the real component of photoconductivity decays rapidly to zero, and the imaginary part is negative—typical signatures of an exciton-dominated system in the quasi-equilibrium state after the initial photoconductivity decay. By contrast, the longest ribbon (58 nm) exhibits a sustained positive real photoconductivity attributable to long-lived free carriers, with little decay evident between 50 and 250 ps.

To quantify the contributions of excitons and free carriers within the overall conductivity, we model the frequency-resolved photoconductivity spectra at various pump-probe delays as a linear contribution from both types: *σ*(*ω*, *t*) *= A*(*t*)*·σ*_F_(*ω*) *+ B*(*t*)*·σ*_EX_(*ω*), where *σ*_F_(*ω*) and *σ*_EX_(*ω*) represent the frequency-dependent free carrier and exciton responses described in Eqs. (1–3), and *A*(*t*) and *B*(*t*) denote their respective time-dependent weights. *σ*_F_(*ω*) and *σ*_EX_(*ω*) are obtained from the response at early times (~0.5 ps) and from the exciton-dominated temporal region (see Supplementary Fig. 4 for an example), respectively. By applying the model, we determined the time-dependent exciton fraction *η*, defined as *η* = *B*(*t*) / [*A*(*t*) *+ B*(*t*)], as summarized in Fig. 3b for all three AHM-GNR samples. Our results show that, for short ribbons (6 and 11 nm), excitons rapidly become the dominant species within 10 ps after excitation. In contrast, long ribbons (58 nm) retain a significant population of long-lived free carriers, with about 30% of the carriers remaining unbound even after 10 ps. Across all samples, this composition stabilizes on the picosecond timescale and remains nearly constant up to 1 nanosecond (ns). We note that, within the sub-ns window, the radiative recombination can be a significant competing channel in many one-dimensional nanostructures. Previous time-resolved photoluminescence measurements on identical 11 nm AHM-GNR dispersions reported an exciton radiative timescale up to ~8 ns^11^. Given that this radiative decay timescale is substantially longer than our maximum observation window (~1 ns) and the sub-ps exciton dissociation processes, we expect that radiative losses play a relatively minor role in the early-time carrier and exciton dynamics discussed here.

The long-lived free carrier states observed in the 58 nm AHM-GNR sample are particularly promising for optoelectronic applications, such as photodetectors and photovoltaics, where efficient exciton splitting is necessary for high performance. While entropy-driven free carrier generation (as described by the Saha model) might explain such behavior at low excitation densities^55^, our data exclude this mechanism, as nearly all carriers become excitons even in the longest ribbons under our experimental conditions (see Supplementary Note 3 and Supplementary Fig. 5). Instead, we attribute the length-dependent generation of free carriers in GNRs to a kinetic process governed by the interplay between ribbon length and the effective spatial range of Coulomb attraction. The Bjerrum length *R*_B_ = *e*^2^/(4*πε*_0_*ε*_r_*k*_B_*T*)^56–58^ defines the distance where thermal energy matches the Coulomb potential between carriers, where *ε*_r_, *k*_B_, and *T* represent the relative permittivity of the solvent, the Boltzmann constant, and the temperature, respectively. We note that charge carriers in GNRs are confined to a 1D geometry, resulting in strong excitonic effects^11^. As such, in the early timescale following optical excitation, excitation above the bandgap generates transient hot free carriers, and only those with sufficiently high kinetic energy can effectively overcome the strong short-range 1D Coulomb potential for charge separation: greater photon energies yield excess kinetic energy, allowing electrons and holes to separate more effectively. Following this initial dissociation process, however, the decisive criterion for generating long-lived free carriers is whether they can separate further before cooling to a distance at which the remaining, solvent‑screened Coulomb attraction drops below *k*_B_*T*. Moreover, the GNRs are narrow but embedded in a liquid solvent with a comparable or higher dielectric constant. Under these conditions, the short-range excitonic physics is governed by the quasi-1D, strongly bound regime, whereas at larger separations the Coulomb interaction is expected to be dominated by solvent screening and can be reasonably described by a 3D-screened potential^59^. Consequently, we estimate *R*_B_ using the solvent-screened 3D Coulomb interaction rather than a strictly 1D potential. This length-dependent separation is closely related to the HED mechanism known from semiconducting polymer heterostructures^60,61^, but here it occurs within a single GNR without the need for interfaces or charge-transfer states. Efficient HED in GNRs relies on excess energy and high charge carrier mobility, particularly of hot charge carriers, to enable separation before rapid exciton formation. Indeed, the high charge carrier mobility in GNRs could balance the large excitonic effect, leading to efficient free carrier generation by HED. In estimating this mobility, we adopt the reported effective mass (m^*^ $\approx$ 0.08 *m*_0_)^43^, as a common value for the three GNRs studied here. It is well established that in GNRs, the carrier effective mass is primarily determined by ribbon width and edge geometry, which dictate the band-edge dispersion^62,63^. When increasing the length of GNRs, bandgap saturation occurs once the length exceeds ~5-8 nm (depending on the ribbon structure)^37–39^. Since all three GNR lengths exhibit optical bandgap saturation, we do not expect the effective mass to show a significant length dependence. Combined with the measured scattering time (*τ* ≈ 28 fs), we estimate a short-range charge carrier mobility (*μ*) in our GNRs to be up to ~ 650 cm^2^·V^−1^·s^−1^ using the expression as *μ* = e‧τ / m^*^, where *e* is the elementary charge. This mobility value is inferred from THz photoconductivity and thus represents short-range, high-frequency charge transport at the nanometer length scale, rather than macroscopic device mobilities.

For short GNRs (e.g., *l* < 2*R*_B_), the spatial limitation imposed by the ribbon length ensures almost all carriers become excitons within ~ 10 ps. For longer ribbons (*l* > 2*R*_B_), electrons can travel far enough from their holes to escape their Coulomb potential, resulting in long-lived free charges. This separation between electrons and holes is maintained until the 1D random-walk diffusion returns them within a distance of *R*_B_ from each other, at which point exciton formation can occur. Using the static dielectric constant of toluene, we calculate *R*_B_ to be approximately 20 nm. This value aligns well with our experimental observation that HED emerges for GNR lengths between 11 and 58 nm. The agreement between the calculated *R*_B_ and the experimental results supports our proposed mechanism. Notably, *R*_B_ extends far beyond conventional quantum confinement (represented by the Bohr radius of roughly 1 nm), offering a more accurate framework for understanding charge separation in GNRs. In contrast, the equilibrium exciton size remains much smaller, at approximately 0.7 nm, due to the localized nature of excitons in these systems^11^. Despite this strongly bound nature, we estimate the exciton spatial extent (polarizable length) to be ~1.1 nm, derived from the exciton polarizability (see Supplementary Fig. 6). This extension exceeds the local atomic-scale width fluctuations (<0.7 nm). As a result, the exciton wavefunction effectively averages over these short-range variations, preventing strong exciton localization by sub-nanometer geometric defects.

### Band dispersion-dependent HED in GNRs

Our study on the role of the GNR length in tuning HED offers crucial insights into the kinetic escape probability of hot carriers and its impact on carrier dissociation efficiency. Within this framework, besides the ribbon length, another key factor is the transient charge carrier mobility in GNRs: a high charge carrier mobility favors long-distance transport within the brief (sub-ps) time window for escape. Assuming that the quasi-steady state mobility also reflects the mobility of hot carriers, we know the charge carrier mobility, *μ*, to be proportional to the ratio of *τ* / m^*^, where m^*^ is the charge carrier effective mass. Since previous studies have found that the scattering time *τ* in GNRs was largely independent of ribbon structure (typically 30-50 fs)^43^, we anticipate that m^*^ or intrinsic electronic structures of GNRs should play a profound role in determining HED efficiency: explicitly, the HED efficiency in GNRs should increase with decreasing m^*^.

**Fig. 4:**
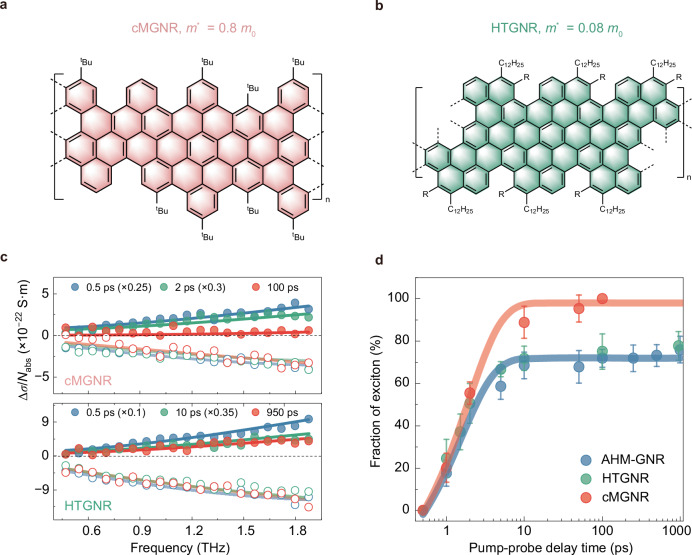
Band dispersion-dependent long-lived free carrier generation in GNRs. **a**, **b** Molecular structure of cMGNR and HTGNR, respectively. **c** Complex frequency-resolved THz photoconductivity of cMGNR and HTGNR at the indicated delay times after the maximum THz photoconductivity following 3.1 eV excitation. The amplitudes of real (solid dots) and imaginary (open dots) parts of photoconductivity at ~0.5 ps and ~2 ps are scaled by the indicated factors for visual comparison. The solid lines are fitted to the model described in the main text for photoconductivity cuts. **d** The relative percentage of excitons as a function of the pump-probe delay time for three GNRs. The error bars are calculated via the standard error propagation from the uncertainties of *A*(*t*) and *B*(*t*). The solid lines represent fits to a single exponential rise function.

To test this hypothesis, we synthesized two additional long nanoribbons: cMGNR (*l* = 70 nm)^64^ and HTGNR (*l* = 43 nm), and determined their HED efficiency following photoexcitation. Figures 4a, b show the molecular structure and effective mass of cMGNR and HTGNR materials, respectively. The cMGNR incorporates *tert*-butyl (^t^Bu) side groups along a curved multi-edged backbone composed of alternating cove, armchair, and gulf segments. This hybrid edge configuration induces a slightly nonplanar geometry and flattens both the valence and conduction bands, resulting in relatively heavy carriers with m^*^ of ~0.8 *m*_0_^64^. In contrast, the HTGNR is achieved by integrating *tert*-butylphenyl substituents at the cove-edge positions of the pristine periodic cove-zigzag-edged GNR (6-CZGNR-(2,1)), which exhibits a much lighter m^*^ of about 0.08 *m*_0_. These structural and electronic distinctions provide a useful framework to examine how band dispersion and carrier mobility dictate intra-ribbon HED. The time-resolved photoconductivity dynamics of cMGNR and HTGNR dispersions after excitation at above-*E*_g_ (3.1 eV) and near-*E*_g_ energies are shown in Supplementary Fig. 7 and Supplementary Fig. 8. In Fig. 4c and Supplementary Fig. 9, we present the frequency-dependent photoconductivity at different time cuts for two GNRs, and show the inferred fractional exciton population in Fig. 4d. As expected, in cMGNR (with a relatively large m^*^ = 0.8 *m*_0_), the majority of the charge carriers condense into exciton states within ~10 ps. In contrast, HTGNR and AHM-GNR (both with m^*^ = 0.08 *m*_0_) maintain a free carrier fraction of about 30%. To explain these observations, we estimate a lower bound for carrier mobility required for efficient HED – approximately 600 cm^2^‧V^−1^‧s^−1^ – based on the diffusion length scale of *R*_B_ (~ 20 nm) and the transient exciton dissociation timescale (~ 250 fs, corresponding to the rise time in the photoconductivity dynamics). This threshold mobility reflects that HED is not solely determined by the available excess energy or geometric length scale, but is fundamentally constrained by the material’s band structure and transient carrier mobility. We note that, in the HED process, the initial non-equilibrium charge carrier mobility can be very high. However, the quasi-equilibrium mobility after ~ps carrier relaxation may be dominated by polaron formation, which significantly reduces carrier mobility. The formation of polarons has been experimentally demonstrated in GNRs^65,66^ and may facilitate the long-lived charge-separated states observed in our longest ribbons.

## Discussion

This work establishes a physical framework for understanding how spatial confinement, carrier mass, and excitation energy jointly govern the dissociation of strongly bound excitons in one-dimensional systems. While the classical Onsager model explains field-assisted exciton separation in bulk materials, its applicability to low-dimensional systems remains uncertain. In particular, in systems without external fields, whether and how tightly bound excitons can dissociate to produce long-lived mobile carriers has been unclear. Our work demonstrates that the excess energy of hot carriers can be seen as an effective field to drive intra-ribbon exciton dissociation, provided two intrinsic criteria are met: a ribbon length exceeding a critical threshold (*l* > 2*R*_B_) and a sufficiently high charge carrier mobility (and thus a low carrier m^*^). This m^*^-dependent dissociation efficiency directly reflects the interplay between electronic band dispersion and exciton dynamics, highlighting a parameter space largely unaccounted for in classical exciton models. Furthermore, although the free carrier generation efficiency by HED is similar in HTGNR and AHM-GNR, their *E*_B_s differ significantly. Photoconductivity measurements quantify the *E*_B_ of HTGNR at 709 ± 49 meV, which is substantially higher than that of AHM-GNRs. This result further indicates that on an ultrafast timescale, the probability of generating free carriers is governed by the initial energetic conditions and local band dispersion, rather than the absolute depth of the Coulomb potential.

Our identification of a critical exciton dissociation length in GNRs offers a unifying explanation for previously reported ultrafast recombination in short ribbons. It provides a clear design principle for achieving free-carrier generation without relying on heterojunctions or built-in fields. These results suggest that the system dimensionality, Coulomb interactions between photogenerated charge carriers, and electronic band structure must be considered jointly when evaluating charge separation efficiency in strongly bound excitonic systems. Finally, the ability to efficiently generate long-lived free carriers in GNRs, as revealed by the critical dependence on ribbon length and band dispersion, is directly relevant for their implementation in optoelectronic devices such as photodetectors, solar cells, and phototransistors. While practical solid-state architectures inevitably introduce substrates and electrical contacts that modify absolute mobility and dielectric screening, our contact-free measurements isolate the intrinsic non-equilibrium processes governing charge separation. These solution-phase measurements effectively provide a fundamental reference point for performance when extrinsic losses are minimized. By exceeding the *R*_B_ threshold and optimizing m^*^, GNRs can sustain free-carrier populations even in strongly excitonic regimes, pointing to strategies to enhance photocurrent and quantum efficiency in real nanoribbon-based device architectures. These insights provide actionable guidance for selecting or engineering GNRs for integration into solid-state device platforms, where overcoming rapid exciton recombination is pivotal for efficient operation.

While the present study establishes the intrinsic physical limits of free-carrier generation in isolated GNRs, transitioning these nanomaterials into practical solid-state architectures necessitates understanding their interaction with the immediate environment. Moving to supported or encapsulated GNR films introduces additional control parameters, including dielectric screening, interfacial disorder, heat-dissipation pathways, and inter-GNR coupling. Environmental screening is known to renormalize Coulomb interactions in low-dimensional systems, thereby substantially modifying exciton binding and charge-separation energetics^67^. Furthermore, substrate coupling can alter cooling and relaxation dynamics, directly dictating whether hot populations can successfully access charge-separated configurations before relaxing to strongly bound states. Therefore, systematic studies of these supported geometries (specifically varying the substrate permittivity and thermal coupling) represent a natural and critical next step toward device integration of GNRs. Such efforts will bridge the gap between intrinsic photophysics and the rational design of optimal device configurations for next-generation photodetectors, solar cells, and phototransistors.

In summary, through a systematic study of the interplay among the excess energy of photogenerated hot carriers, the length and effective mass of GNRs on exciton dissociation, we report intra-GNR hot exciton dissociation as the dominant mechanism for free carrier generation in GNRs. We unveil a critical length scale (*R*_B_ ~ 20 nm) in GNRs, beyond which hot carriers can effectively dissociate spatially to produce extremely long-lived (over ns) free charge carriers with high efficiency. For GNRs with shorter length *l* < 2*R*_B_, the charge separation distance by the HED effect is intrinsically limited by the ribbon length so that the strong electron-hole Coulomb interactions lead to ultrafast exciton formation on the ~ 10 ps timescale. Crucially, we demonstrate that even when GNRs exceed this critical length, the effective mass of charge carriers plays a decisive role in determining their properties: low-effective mass GNRs (e.g., 0.08 *m*_0_) exhibit extended carrier lifetimes (over ns) through enhanced mobility, while high-effective mass counterparts (e.g., 0.8 *m*_0_) suffer rapid recombination. Our results provide fundamental insights into the critical roles of the GNR length, band structure, and the excess energy of charge carriers in free carrier generation in GNRs, which is relevant for their applications in optoelectronics.

## Methods

### Sample preparation

For the preparation of GNR dispersions, GNR powders were dispersed in toluene solvent (≥ 99.8%, analytical reagent grade, Fisher Scientific, UK) at a concentration of 0.5 mg‧mL^−1^. The mixture was stirred at 100 °C for 10 h and subsequently sonicated for 30 min to ensure complete dispersion. The quartz cuvettes (thickness of 2 mm) are sequentially cleaned by ultrasonication in deionized water, ethanol, acetone, and toluene (2 min each), followed by drying under a nitrogen stream. The purified GNR solution was then transferred into the quartz cuvettes for Ultraviolet-visible-near-infrared (UV-vis-NIR) absorption and THz spectroscopy measurements.

### UV-vis-NIR absorption measurement

The UV-vis-NIR absorption spectra for AHM-GNR solutions were obtained on HITACHI U-4100 spectrophotometer.

### Optical pump-terahertz probe spectroscopy (OPTP)

Transient photoconductivity of GNRs solutions is investigated using a home-built optical pump-THz probe setup. The ultrafast laser source is a commercial regeneratively amplified Ti:sapphire system, delivering the pulses centered at ~800 nm with a repetition rate of 1 kHz and a duration of ~50 fs. A portion of the output is delivered to a 1-mm-thick (110)-oriented ZnTe crystal for generating the single-cycle THz radiation by optical rectification, while the THz detection is achieved in a second ZnTe crystal through electro-optic sampling, enabling access to a spectral window of 0.4 ~ 2.0 THz. The temporal profile of the THz waveform is measured by scanning the relative delay between the probe and a synchronized 800-nm sampling pulse derived from the same amplifier. For optical excitation, the excitation pulse of 800 nm is routed from the output of the laser, and the pump excitation at 400 nm (~50 fs) is produced by the frequency doubling the 800 nm beam in a *β*-barium borate crystal. Additional pump wavelengths are generated using a commercial optical parametric amplifier (Light Conversion). Pump-induced changes in the transmitted THz field are recorded at both the peak and the first zero-crossing point of the reference THz waveform. Variations at the THz peak amplitude provide direct information about the real part of the photoconductivity, whereas shifts at the zero-crossing reflect the imaginary part. The relative delay between pump and probe pulses is varied with a motorized delay stage to capture the ultrafast evolution of the photoinduced conductivity. Full frequency-dependent spectra are obtained by Fourier transforming the time-domain THz waveform. GNRs solution samples are placed in 2 mm-thick quartz cuvettes. Continuous stirring is employed throughout the measurements to prevent aggregation or sedimentation of the nanoribbons. All experiments are performed under a dry nitrogen atmosphere to minimize absorption of THz radiation by water vapor.

## Supplementary information

Supplementary Information

Transparent Peer Review file

## Data Availability

All the data supporting the findings and mentioned in this study are included in the Article and its Supplementary Information file. The data are available from the corresponding author upon request.

## References

[1] Chen, H. et al. Organic solar cells with 20.82% efficiency and high tolerance of active layer thickness through crystallization sequence manipulation. *Nat. Mater.* **24**, 444–453 (2025). 39824965 10.1038/s41563-024-02062-0

[2] Jiang, Y. et al. Non-fullerene acceptor with asymmetric structure and phenyl-substituted alkyl side chain for 20.2% efficiency organic solar cells. *Nat. Energy* **9**, 975–986 (2024).

[3] Xie, B. et al. Self-filtering narrowband high performance organic photodetectors enabled by manipulating localized Frenkel exciton dissociation. *Nat. Commun.* **11**, 2871 (2020). 32514001 10.1038/s41467-020-16675-x PMC7280211

[4] Lan, Z. et al. Near-infrared and visible light dual-mode organic photodetectors. *Sci. Adv.* **6**, eaaw8065 (2020). 32064330 10.1126/sciadv.aaw8065 PMC6994203

[5] Li, T. et al. Sensitive photodetection below silicon bandgap using quinoid-capped organic semiconductors. *Sci. Adv.* **9**, eadf6152 (2023). 36989368 10.1126/sciadv.adf6152 PMC10058242

[6] Che, B. et al. Post-deposition treatment of Sb_2_Se_3_ enables defect passivation and increased carrier transport dimension for efficient solar cell application. *Angew. Chem. Int. Ed.* **64**, e202425639 (2025). 10.1002/anie.202425639 39921486

[7] Ye, S. et al. Expanding the low-dimensional interface engineering toolbox for efficient perovskite solar cells. *Nat. Energy* **8**, 284–293 (2023).

[8] Li, Z., Yan, T. & Fang, X. Low-dimensional wide-bandgap semiconductors for UV photodetectors. *Nat. Rev. Mater.* **8**, 587–603 (2023).

[9] Qian, Y. et al. Computation-based regulation of excitonic effects in donor-acceptor covalent organic frameworks for enhanced photocatalysis. *Nat. Commun.* **14**, 3083 (2023). 37248231 10.1038/s41467-023-38884-w PMC10227069

[10] Shang, S. et al. A one-dimensional conductive metal-organic framework with extended π-d conjugated nanoribbon layers. *Nat. Commun.* **13**, 7599 (2022). 36494377 10.1038/s41467-022-35315-0 PMC9734122

[11] Tries, A. et al. Experimental observation of strong exciton effects in graphene nanoribbons. *Nano Lett.* **20**, 2993–3002 (2020). 32207957 10.1021/acs.nanolett.9b04816 PMC7311082

[12] Yang, L., Cohen, M. L. & Louie, S. G. Excitonic effects in the optical spectra of graphene nanoribbons. *Nano Lett.* **7**, 3112–3115 (2007). 17824720 10.1021/nl0716404

[13] Miao, Z. et al. Organic light-emitting transistors with high efficiency and narrow emission originating from intrinsic multiple-order microcavities. *Nat. Mater.* **24**, 917–924 (2025). 40155555 10.1038/s41563-025-02191-0

[14] Hasan, M. et al. Probing polaron-induced exciton quenching in TADF based organic light-emitting diodes. *Nat. Commun.* **13**, 254 (2022). 35017481 10.1038/s41467-021-27739-x PMC8752634

[15] Han, Z. et al. Tightly bonded excitons in chiral metal clusters for luminescent brilliance. *Nat. Commun.* **16**, 1–12 (2025). 39984514 10.1038/s41467-025-57209-7 PMC11845751

[16] Hendry, E., Schins, J. M., Candeias, L. P., Siebbeles, L. D. A. & Bonn, M. Efficiency of exciton and charge carrier photogeneration in a semiconducting polymer. *Phys. Rev. Lett.* **92**, 196601 (2004). 15169428 10.1103/PhysRevLett.92.196601

[17] Arkhipov, V. I., Emelianova, E. V. & Bässler, H. Hot exciton dissociation in a conjugated polymer. *Phys. Rev. Lett.* **82**, 1321–1324 (1999).

[18] Yang, Y., Rodríguez-Córdoba, W. & Lian, T. Multiple exciton generation and dissociation in PbS quantum dot-electron acceptor complexes. *Nano Lett.* **12**, 4235–4241 (2012). 22757981 10.1021/nl301847r

[19] Bacher, G. et al. Biexciton versus exciton lifetime in a single semiconductor quantum dot. *Phys. Rev. Lett.* **83**, 4417–4420 (1999).

[20] Schaller, R. D. & Klimov, V. I. High efficiency carrier multiplication in PbSe nanocrystals: implications for solar energy conversion. *Phys. Rev. Lett.* **92**, 186601 (2004). 15169518 10.1103/PhysRevLett.92.186601

[21] Massicotte, M. et al. Dissociation of two-dimensional excitons in monolayer WSe_2_. *Nat. Commun.* **9**, 1633 (2018). 29691376 10.1038/s41467-018-03864-y PMC5915447

[22] Handa, T. et al. Spontaneous exciton dissociation in transition metal dichalcogenide monolayers. *Sci. Adv.* **10**, eadj4060 (2024). 38295176 10.1126/sciadv.adj4060 PMC10830119

[23] He, K. et al. Tightly bound excitons in monolayer WSe_2_. *Phys. Rev. Lett.* **113**, 026803 (2014). 25062219 10.1103/PhysRevLett.113.026803

[24] Chernikov, A. et al. Exciton binding energy and nonhydrogenic Rydberg series in monolayer WS_2_. *Phys. Rev. Lett.* **113**, 076802 (2014). 25170725 10.1103/PhysRevLett.113.076802

[25] Kaiser, V., Bramwell, S. T., Holdsworth, P. C. W. & Moessner, R. Onsager’s Wien effect on a lattice. *Nat. Mater.* **12**, 1033–1037 (2013). 23934036 10.1038/nmat3729

[26] Cao, T., Zhao, F. & Louie, S. G. Topological phases in graphene nanoribbons: junction states, spin centers, and quantum spin chains. *Phys. Rev. Lett.* **119**, 076401 (2017). 28949674 10.1103/PhysRevLett.119.076401

[27] Niu, W. et al. Exceptionally clean single-electron transistors from solutions of molecular graphene nanoribbons. *Nat. Mater.* **22**, 180–185 (2023). 36732344 10.1038/s41563-022-01460-6 PMC10208969

[28] Rizzo, D. J. et al. Topological band engineering of graphene nanoribbons. *Nature* **560**, 204–208 (2018). 30089918 10.1038/s41586-018-0376-8

[29] Slota, M. et al. Magnetic edge states and coherent manipulation of graphene nanoribbons. *Nature* **557**, 691–695 (2018). 29849157 10.1038/s41586-018-0154-7

[30] Denk, R. et al. Exciton-dominated optical response of ultra-narrow graphene nanoribbons. *Nat. Commun.* **5**, 4253 (2014). 25001405 10.1038/ncomms5253

[31] Senkovskiy, B. V. et al. Spectroscopic characterization of N = 9 armchair graphene nanoribbons. *Phys. Status Solidi RRL* **11**, 1700157 (2017).

[32] Prezzi, D., Varsano, D., Ruini, A., Marini, A. & Molinari, E. Optical properties of graphene nanoribbons: The role of many-body effects. *Phys. Rev. B* **77**, 041404 (2008).

[33] Dubey, R. K., Melle-Franco, M. & Mateo-Alonso, A. Twisted molecular nanoribbons with up to 53 linearly-fused rings. *J. Am. Chem. Soc.* **143**, 6593–6600 (2021). 33876941 10.1021/jacs.1c01849

[34] Dubey, R. K. et al. Accelerated iterative synthesis of ultralong graphene nanoribbons with full atomic precision. *Chem* **9**, 2983–2996 (2023).

[35] Hernández-Culebras, F., Melle-Franco, M. & Mateo-Alonso, A. Doubling the length of the longest pyrene-pyrazinoquinoxaline molecular nanoribbons. *Angew. Chem.* **134**, e202205018 (2022). 10.1002/anie.202205018 PMC9321727 35467070

[36] Gröning, O. et al. Engineering of robust topological quantum phases in graphene nanoribbons. *Nature* **560**, 209–213 (2018). 30089919 10.1038/s41586-018-0375-9

[37] Kimouche, A. et al. Ultra-narrow metallic armchair graphene nanoribbons. *Nat. Commun.* **6**, 10177 (2015). 26658960 10.1038/ncomms10177 PMC4682157

[38] Talirz, L. et al. Band gap of atomically precise graphene nanoribbons as a function of ribbon length and termination. *ChemPhysChem* **20**, 2348–2353 (2019). 31304992 10.1002/cphc.201900313

[39] Drummer, M. C. et al. Long-lived excited state in a solubilized graphene nanoribbon. *J. Phys. Chem. C.* **126**, 1946–1957 (2022).

[40] Wang, X. et al. Cove-edged graphene nanoribbons with incorporation of periodic zigzag-edge segments. *J. Am. Chem. Soc.* **144**, 228–235 (2022). 34962807 10.1021/jacs.1c09000

[41] Jensen, S. A. et al. Ultrafast photoconductivity of graphene nanoribbons and carbon nanotubes. *Nano Lett.* **13**, 5925–5930 (2013). 24093134 10.1021/nl402978s

[42] Huang, Y. et al. Intrinsic properties of single graphene nanoribbons in solution: synthetic and spectroscopic studies. *J. Am. Chem. Soc.* **140**, 10416–10420 (2018). 30084630 10.1021/jacs.8b06028 PMC6643163

[43] Ivanov, I. et al. Role of edge engineering in photoconductivity of graphene nanoribbons. *J. Am. Chem. Soc.* **139**, 7982–7988 (2017). 28525278 10.1021/jacs.7b03467

[44] Hendry, E. et al. Interchain effects in the ultrafast photophysics of a semiconducting polymer: THz time-domain spectroscopy of thin films and isolated chains in solution. *Phys. Rev. B* **71**, 125201 (2005).

[45] Wang, F. et al. Exciton polarizability in semiconductor nanocrystals. *Nat. Mater.* **5**, 861–864 (2006). 17028577 10.1038/nmat1739

[46] Cocker, T. L. et al. Microscopic origin of the Drude-Smith model. *Phys. Rev. B* **96**, 205439 (2017).

[47] Smith, N. Classical generalization of the Drude formula for the optical conductivity. *Phys. Rev. B* **64**, 155106 (2001).

[48] Zheng, W., Zorn, N. F., Bonn, M., Zaumseil, J. & Wang, H. I. Probing carrier dynamics in sp^3^-functionalized single-walled carbon nanotubes with time-resolved terahertz spectroscopy. *ACS Nano* **16**, 9401–9409 (2022). 35709437 10.1021/acsnano.2c02199 PMC9246260

[49] Yao, X. et al. Synthesis of nonplanar graphene nanoribbon with Fjord edges. *J. Am. Chem. Soc.* **143**, 5654–5658 (2021). 33825484 10.1021/jacs.1c01882 PMC8154539

[50] Chen, Z. et al. Chemical vapor deposition synthesis and terahertz photoconductivity of low-band-gap N = 9 armchair graphene nanoribbons. *J. Am. Chem. Soc.* **139**, 3635–3638 (2017). 28248492 10.1021/jacs.7b00776

[51] He, X. et al. N-doped nonalternant nanoribbons with up to 29 linearly-fused rings and high charge-carrier mobilities. *Angew. Chem. Int. Ed.* **64**, e202514214 (2025). 10.1002/anie.202514214 40838469

[52] Ulbricht, R., Hendry, E., Shan, J., Heinz, T. F. & Bonn, M. Carrier dynamics in semiconductors studied with time-resolved terahertz spectroscopy. *Rev. Mod. Phys.* **83**, 543–586 (2011).

[53] Hadipour, H. et al. Screening of long-range Coulomb interaction in graphene nanoribbons: armchair versus zigzag edges. *Phys. Rev. B* **98**, 205123 (2018).

[54] Narita, A. et al. Synthesis of structurally well-defined and liquid-phase-processable graphene nanoribbons. *Nat. Chem.* **6**, 126–132 (2014). 24451588 10.1038/nchem.1819

[55] Kaindl, R. A., Hägele, D., Carnahan, M. A. & Chemla, D. S. Transient terahertz spectroscopy of excitons and unbound carriers in quasi-two-dimensional electron-hole gases. *Phys. Rev. B* **79**, 045320 (2009).

[56] Haghighat, S., Ostresh, S. & Dawlaty, J. M. Controlling proton conductivity with light: a scheme based on photoacid doping of materials. *J. Phys. Chem. B* **120**, 1002–1007 (2016). 26771862 10.1021/acs.jpcb.6b00370

[57] Moritz, R. et al. Ion size approaching the Bjerrum length in solvents of low polarity by dendritic encapsulation. *Macromolecules* **47**, 191–196 (2014).

[58] Kavokine, N., Marbach, S., Siria, A. & Bocquet, L. Ionic Coulomb blockade as a fractional Wien effect. *Nat. Nanotechnol.* **14**, 573–578 (2019). 30962547 10.1038/s41565-019-0425-y

[59] Villegas, C. E. P. & Rocha, A. R. Screened hydrogen model of excitons in semiconducting nanoribbons. *Phys. Rev. B* **109**, 165425 (2024).

[60] Jailaubekov, A. E. et al. Hot charge-transfer excitons set the time limit for charge separation at donor/acceptor interfaces in organic photovoltaics. *Nat. Mater.* **12**, 66–73 (2013). 23223125 10.1038/nmat3500

[61] Grancini, G. et al. Hot exciton dissociation in polymer solar cells. *Nat. Mater.* **12**, 29–33 (2013). 23223127 10.1038/nmat3502

[62] Son, Y.-W., Cohen, M. L. & Louie, S. G. Energy gaps in graphene nanoribbons. *Phys. Rev. Lett.* **97**, 216803 (2006). 17155765 10.1103/PhysRevLett.97.216803

[63] Tran, V. & Yang, L. Scaling laws for the band gap and optical response of phosphorene nanoribbons. *Phys. Rev. B* **89**, 245407 (2014).

[64] Yang, L. et al. Solution synthesis and characterization of a long and curved graphene nanoribbon with hybrid cove-armchair-gulf edge structures. *Adv. Sci.* **9**, 2200708 (2022). 10.1002/advs.202200708 PMC9259722 35322602

[65] Lindenthal, S. et al. Understanding the optical properties of doped and undoped 9-armchair graphene nanoribbons in dispersion. *ACS Nano* **17**, 18240–18252 (2023). 37695780 10.1021/acsnano.3c05246 PMC10540269

[66] Hermosilla-Palacios, M. A. et al. Polaron delocalization and transport in doped graphene nanoribbon thin films. *ACS Nano* **19**, 25732–25743 (2025). 40622767 10.1021/acsnano.5c03888 PMC12291592

[67] Raja, A. et al. Coulomb engineering of the bandgap and excitons in two-dimensional materials. *Nat. Commun.* **8**, 15251 (2017). 28469178 10.1038/ncomms15251 PMC5418602

