# Supplementary material for: Hot exciton dissociation in graphene nanoribbons
Source: Nat Commun. 2026 Jun 12;17:5260. doi: 10.1038/s41467-026-74158-x (PMC13263337; doi:10.1038/s41467-026-74158-x)
Supplement: Supplementary file 1 — Supplementary Information [file 41467_2026_74158_MOESM1_ESM.pdf]

Supporting Information for

Hot Exciton Dissociation in Graphene Nanoribbons

Guanzhao Wen[1#], Fugui Xu[2#], Alexander Tries[1,3#§], Wenhao Zheng[1], Lucia Di Virgilio[1], Shuai Fu[1], Xinyu Chen[4], Lin Yang[5], Zijie Xiao[1], Mathias Kläui[3], Silvio Osella[6], Ji Ma[5,7], Xu Wang[4], Xinliang Feng[5,8], Yiyong Mai[2], Mischa Bonn[1*], and Hai I. Wang[1,9*]

[1] Max Planck Institute for Polymer Research, Ackermannweg 10, 55128 Mainz, Germany

[2] State Key Laboratory of Synergistic Chem-Bio Synthesis, School of Chemistry and Chemical Engineering, Shanghai Key Laboratory of Electrical Insulation and Thermal Ageing, Shanghai Jiao Tong University, 800 Dongchuan RD, 200240 Shanghai, China

[3] Institute of Physics, Johannes Gutenberg-University Mainz, Staudingerweg 7, 55128 Mainz, Germany

[4] College of Polymer Science and Engineering, State Key Laboratory of Advanced Polymer Materials, Sichuan University, 610065 Chengdu, China

[5] Max Planck Institute of Microstructure Physics, Weinberg 2, 06120 Halle, Germany

[6] Materials and Processes Simulation Lab, Centre of New Technologies, University of Warsaw, Bana-cha 2C, 02-097 Warsaw, Poland

[7] Beijing National Laboratory for Molecular Sciences, CAS Key Laboratory of Organic Solids, Institute of Chemistry, Chinese Academy of Sciences, 100190 Beijing, China

[8] Centre for Advancing Electronics Dresden & Faculty of Chemistry and Food Chemistry, Technische Universität Dresden, 01062 Dresden, Germany

[9] Nanophotonics, Debye Institute for Nanomaterials Science, Utrecht University, Princetonplein 1, 3584 CC Utrecht, the Netherlands

[§] Present address: Bundesdruckerei GmbH, Kommandantenstraße 18, 10969 Berlin, Germany

[#] These authors contributed equally

[*]E-mail: bonn@mpip-mainz.mpg.de; h.wang5@uu.nl

Contents

Supplementary Notes 3

Supplementary Note 1. Calculation of Absorbed Photon Density 3

Supplementary Note 2. Extraction of Frequency-Resolved Photoconductivity ... 4

Supplementary Note 3. Entropy-Driven Free carriers Generation 5

Supplementary Figures and Tables 6

Supplementary Figure 1. Representative pump-fluence dependence of the peak THz photoconductivity. 6

Supplementary Figure 2. Representative pump-fluence dependence of normlized time-resolved THz photoconductivity. 7

Supplementary Figure 3. The frequency-resolved complex photoconductivity spectra of AHM-GNR solution with different lengths 7

Supplementary Figure 4. Frequency-dependent complex photoconductivity spectra of the AHM-GNR (58 nm) dispersion 8

Supplementary Figure 5. Exciton fraction as a function of total photon excitation density.. 8

Supplementary Figure 6. Extraction of the polarizability for the 11 nm AHM-GNR.. 9

Supplementary Figure 7. The excitation pump fluence-dependent normalized time-resolved and the corresponding peak THz photoconductivity for HTGNR dispersion and cMGNR dispersion 10

Supplementary Figure 8. Long time-scale time-resolved photoconductivity of HTGNR 11

Supplementary Figure 9. Frequency-dependent complex photoconductivity spectra of HTGNR and cMGNR dispersions 11

Supplementary Table 1. Sample information of three AHM-GNR with different lengths.. 12

Supplementary Table 2. The detailed Drude-Smith model fitting parameters of the complex photoconductivity of AHM-GNR solutions 12

Supplementary References 13

Supplementary Notes

Supplementary Note 1. Calculation of Absorbed Photon Density

The absorbed photon density N_{abs} of GNR dispersions at a given excitation pump energy is determined to quantify the number of photons absorbed per unit area for each excitation pulse. It is obtained as $N_{abs} = N_{photon} \cdot (1 - 10^{-A})$, where A represents the optical absorbance of the GNR dispersions at the excitation wavelength. The incident photon density N_{photon} is derived from the laser parameters according to

$$N_{photon}\ (m^{-2}) = \frac{E_{pulse}\lambda}{hc} = \frac{P_{avg}}{f_{rep} \cdot \frac{hc}{\lambda} \cdot \pi r^2} \qquad (S1)$$

where, P_{avg} is the average laser power incident on the dispersion (in W), f_{rep} is the pulse repetition frequency (500 Hz after the chopper), and h is Planck's constant (6.626×10^{-34} J·s), and c is the speed of light in vacuum (3.0×10^{8} m·s^{-1}), and λ is the excitation wavelength (in m), and r is the pump beam radius at the sample position. The term $E_{pulse} = P_{avg} / f_{rep}$ corresponds to the energy per laser pulse, while hc / λ represents the energy of a single photon. The factor $(1 - 10^{-A})$ represents the fraction of photons actually absorbed by the sample. This formulation provides an estimation of the absorbed photon density per pulse, which is essential for evaluating the excitation fluence and correlating it with the photoconductivity extracted from Terahertz measurement.

Supplementary Note 2. Extraction of Frequency-Resolved Photoconductivity

To investigate the intrinsic carriers transport properties in GNR dispersions, we performed the optical pump-THz probe time domain spectroscopy. In detail, the entire transmitted THz waveform of sample without ($E_0(t)$) and with ($E_P(t)$) optical excitations at a given pump-probe delay time were record based on electrooptical sampling method. After the Fourier transformation, the relative change of transmitted THz waveform ($\Delta E(\omega) = E_P(\omega) - E_0(\omega)$) in frequency domain can be extracted, which is proportional to the change of the complex photoconductivity ($\Delta\sigma(\omega)$).[1]

In our measurement, we conducted the optical pump-THz probe time domain spectroscopy with GNRs solution in toluene. The generated THz field transmits sequentially from the air, the front quartz cuvette window, the solvent, the back quartz cuvette window, and finally the air. The pump pulse excitation profile can be treated as homogeneous when it passes our sample due to the low concentration and absorbance of solution and the larger penetration depth of the excitation pulse. Therefore, the complex conductivity can be extracted as follows:[2–4]

$$\Delta\sigma(\omega) = \left(n^2 - \left(n + \Delta\hat{n}(\omega)\right)^2\right)\frac{i\omega}{Z_0 c} \tag{S2}$$

where $\Delta\hat{n}(\omega)$ is the photo-induced change in the reflective index n, which can be estimated by $\Delta\hat{n}(\omega) = \left(\frac{1}{n}\frac{n_w - n}{n_w + n} + i\frac{\omega d}{c}\right)^{-1}\frac{\Delta E(\omega)}{E_0(\omega)}$, n, n_w, Z_0, d, and c are the real refractive index of the solvent, the real refractive index of the cuvette window, the vacuum impedance, the excitation thickness, and the speed of light, respectively. The parameters used in our analysis are $n = 1.50$ for toluene solvent, $n_w = 2.16$ for the quartz windows.

Supplementary Note 3. Entropy-Driven Free carriers Generation

The bounding electron-hole pair (exciton) can be generated after photoexcitation, which also can be dissociated into the free electron and hole thanks to following Saha's mass action low. This entropy-gain-driven free carrier generation efficiency is closely related to the total photon excitation density (N_{tot}) and exciton binding energy (E_{B}). In detail, the densities of excitons ($\bar{n}_{Ex}$), free electrons ($\bar{n}_e$), free holes ($\bar{n}_h$), and E_{B} following Saha equation,[5] reads as follows:

$$\frac{\bar{n}_e \bar{n}_h}{\bar{n}_{Ex}} = \frac{\mu}{2\pi\hbar^2} k_B T e^{-\frac{E_B}{k_B T}} = S \tag{S3}$$

where μ is the reduced mass of exciton, $\hbar$ is the reduced Planck's constant, $k_{\mathrm{B}}T$ is the thermal energy (25 meV). S represents a parameter for the right-hand side of equation (S3), which become a constant with the given E_{B}, μ and temperature. Considering the exciton fraction $\alpha_{Ex} = \bar{n}_{Ex}/N_{tot} = \bar{n}_{Ex}/(\bar{n}_e + \bar{n}_h)$, the analytical solution of equation (S3) can be expressed as:[6]

$$\alpha_{Ex} \equiv \frac{\bar{n}_{Ex}}{N_{tot}} = 1 + \frac{S}{2N_{tot}} - \sqrt{\left(\frac{S}{2N_{tot}}\right)^2 + \frac{S}{N_{tot}}} \tag{S4}$$

More quantitatively, in this work, the E_{B} of 620 meV for GNR-AHM with 58 nm is used as an example, μ is approximated to the reported value as $0.084 \cdot m_0$[7], and $k_{\mathrm{B}}T$ is 25 meV with the room temperature. Hence, the parameter of S can be calculated to 7.0×10^8 cm^{-2} in this work. Considering the excitation photon density (~ 10^{13}-10^{15} cm^{-2}), the exciton fraction is near 100% even for the longest GNRs, as shown in Figure S2.

Supplementary Figures and Tables

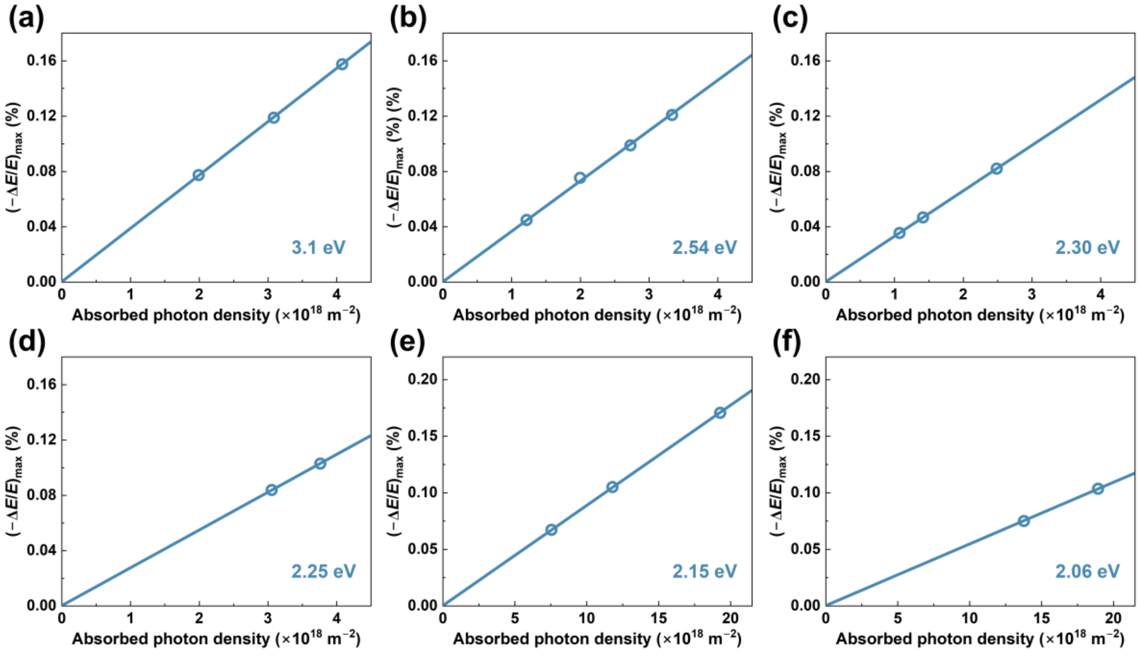

Supplementary Figure 1. Representative pump-fluence dependence of the peak THz photoconductivity. The peak THz signal $(-\Delta E/E)_{max}$ is plotted as a function of the absorbed photon density for (a-f) the 58 nm AHM-GNR dispersion at various excitation photon energies ranging from 2.06 eV to 3.1 eV. The open circles represent experimental data, and the solid lines are the corresponding linear fits.

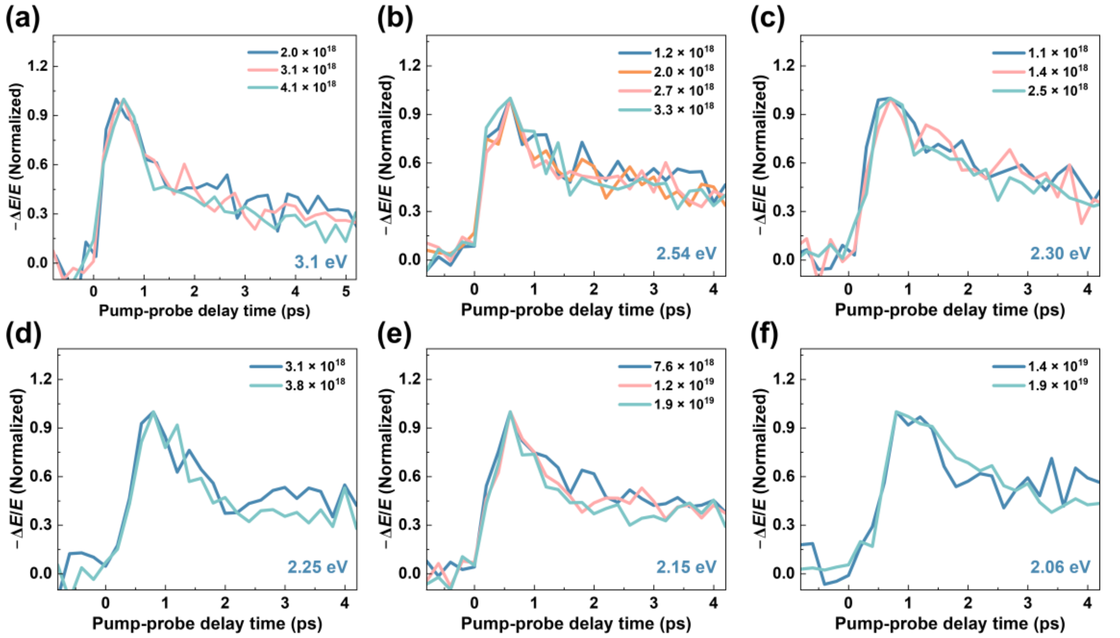

Supplementary Figure 2. Representative pump-fluence dependence of the time-resolved THz photoconductivity. The normalized time-resolved THz photoconductivity is plotted as a function of the absorbed photon density (N_{abs}, m^{-2}) for (a-f) the 58 nm AHM-GNR dispersion at various excitation photon energies ranging from 2.06 eV to 3.1 eV.

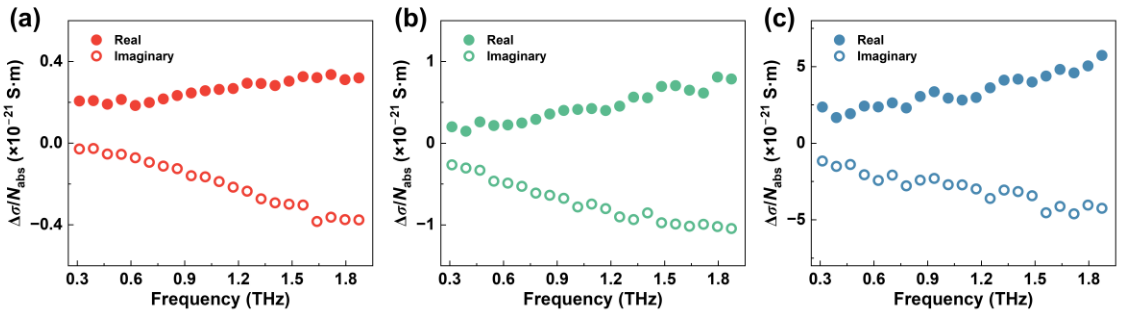

Supplementary Figure 3. Length-dependent complex THz photoconductivity. The frequency-resolved complex photoconductivity spectra (normalized to absorbed photon density) of AHM-GNR solution with lengths of (a) 6 nm, (b) 11 nm, and (c) 58 nm measured at ~0.5 ps after the maximum photoconductivity.

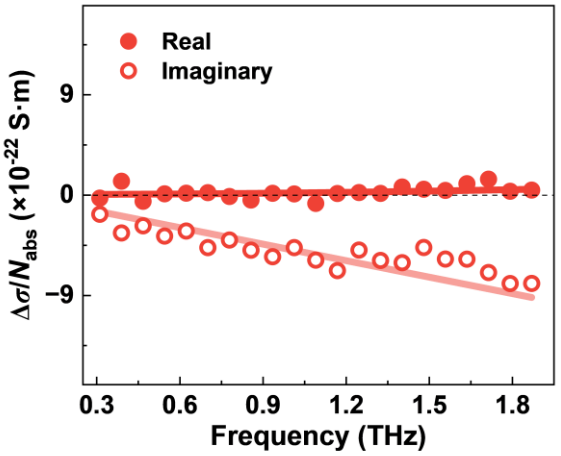

Supplementary Figure 4. Frequency-dependent complex photoconductivity spectra of the AHM-GNR (58 nm) dispersion measured at ~2 ps after the maximum photoconductivity under band-edge energy excitation (~1.62 eV). The solid lines correspond to the Lorentzian model fits (Equation 1 in the main text) used to describe the spectral response. The real part of the conductivity is close to zero, while the imaginary part shows a pronounced dispersive feature, indicating that the photoinduced response at this delay is dominated by excitonic polarization. Therefore, this spectrum is assigned to the exciton-dominated photoconductivity response ($\sigma_{EX}(\omega)$).

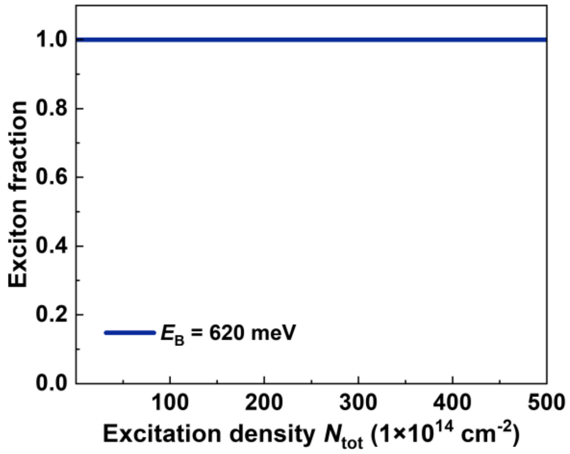

Supplementary Figure 5. Exciton fraction modeled with ~620 meV binding energy. Exciton fraction (α_{EX}) as a function of total photon excitation density (N_{tot}) with the exciton binding energy of 620 meV. The solid line is described by equation (S4).

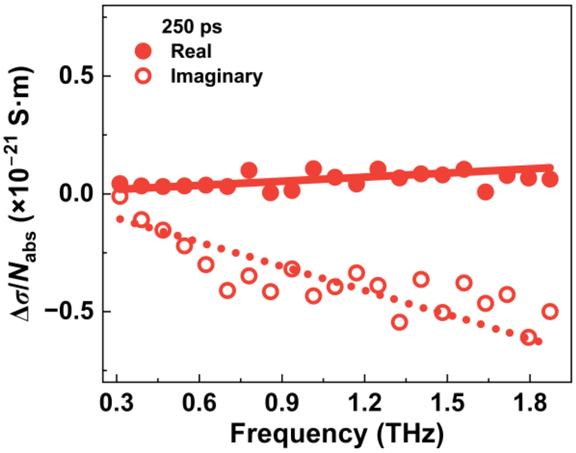

Supplementary Figure 6. Extraction of the polarizability for the 11 nm AHM-GNR. Frequency-dependent complex THz photoconductivity measured at ~250 ps after the maximum photoconductivity. Solid and open circles represent the experimental real and imaginary parts of the photoconductivity, respectively. At this delay time, the frequency dispersion is dominated by bound excitons, which appear as purely polarizable species with a near-zero real component and a negative imaginary component. The solid and dashed lines represent the modeled real and imaginary conductivities, respectively. These theoretical curves are generated by modeling the GNR dispersion as a dielectric composite using the Clausius-Mossotti relation.[8–10] By matching the theoretical conductivity to the experimental data (specifically the linear frequency dependence of the imaginary part), an average exciton polarizability $\alpha \approx 6\ \text{nm}^3$ is extracted, implying an exciton coherence length as ~1.1 nm.

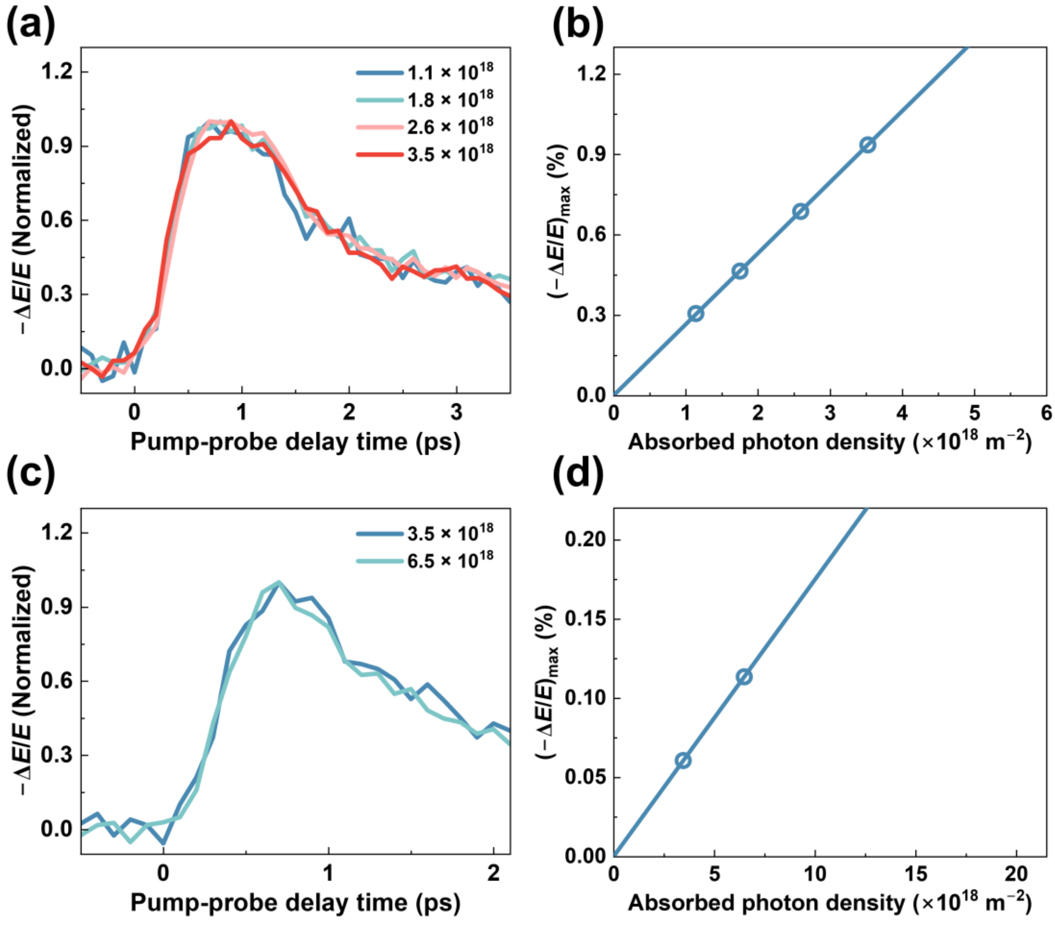

Supplementary Figure 7. Fluence-dependent photoconductivity dynamics. Panels (a) and (c) show the normalized time-resolved THz photoconductivity, while panels (b) and (d) present the corresponding peak photoconductivity $(-\Delta E/E)_{max}$ for (a, b) HTGNR dispersion and (c, d) cMGNR dispersion under the excitation as 3.1 eV. Open circles represent experimental data, and the solid lines in (b) and (d) are linear fits.

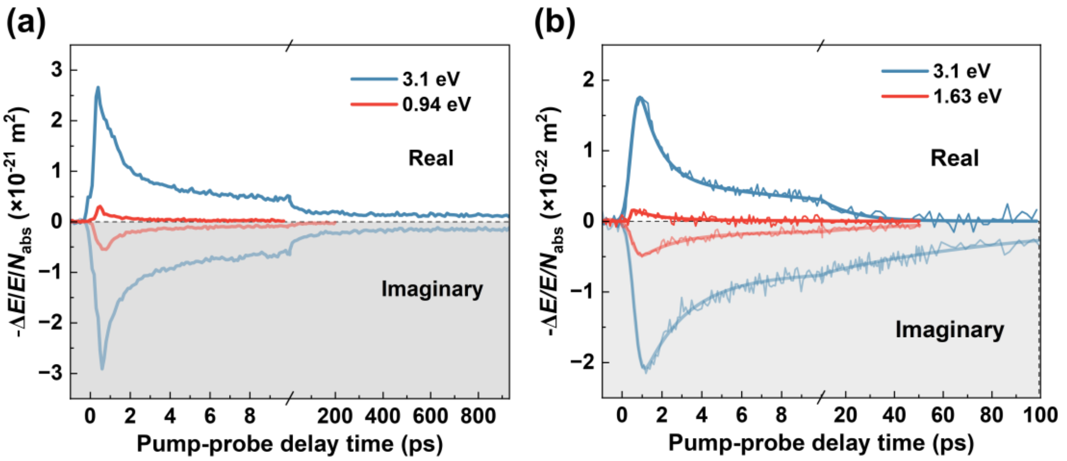

Supplementary Figure 8. Long-lived photoconductivity in cMGNR and HTGNR. Long time-scale time-resolved photoconductivity of (a) HTGNR and (b) cMGNR dispersions excited at above-bandgap (blue solid line) and near-bandgap (red solid line) photon energies.

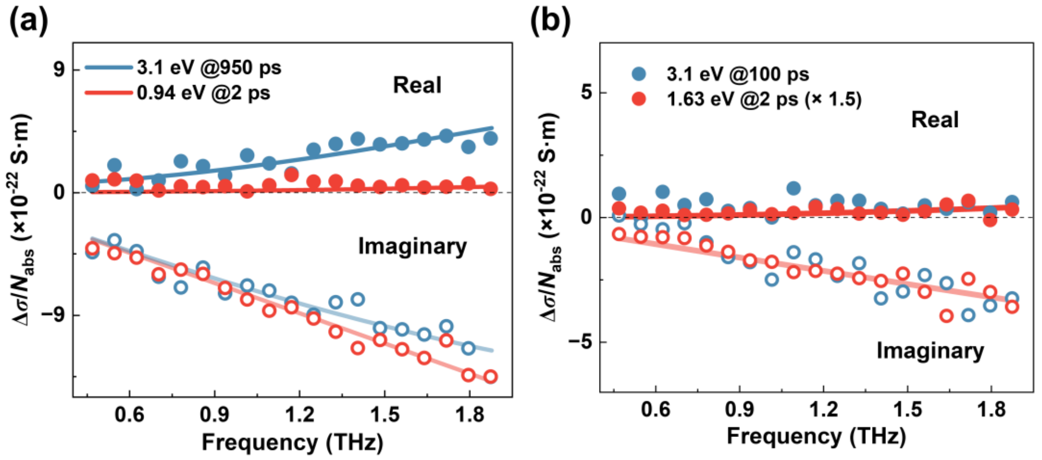

Supplementary Figure 9. Free-carrier and exciton dispersions in GNRs. Frequency-dependent complex photoconductivity spectra of (a) HTGNR and (b) cMGNR dispersions measured at ~2 ps (red dots) and at long delay times (blue dots) after the maximum photoconductivity under near-bandgap (red dots) and above-bandgap (blue dots) excitations, respectively. The red solid lines represent the Lorentzian model fits (Equation 1 in the main text), while the blue solid lines correspond to the fitting curves based on the linear combination of free-carrier and exciton responses as described in the main text.

Supplementary Table 1. Sample information of three AHM-GNR with different lengths.

Sample name		M_n (g/mol)	$Đ$	Length (nm)
AHM-GNR-1	AHM-PP-1	13500	1.40	~6
AHM-GNR-2	AHM-PP-2	23100	1.20	~11
AHM-GNR-3	AHM-PP-3	124000	1.50	~58

The number-average molecular weights of the AHM-PP samples were recorded by gel permeation chromatography (GPC) with a Wyatt Optilab DSP differential refractometer, using tetrahydrofuran (THF) as the eluent and standard polystyrene as the reference.

Supplementary Table 2. The detailed Drude-Smith model fitting parameters of the complex photoconductivity of AHM-GNR solutions (see Figure S1).

AHM-GNR	ω_p (THz)	τ_{DS} (fs)	c
6 nm	2.2 ± 0.04	6.2 ± 0.6	-0.95
11 nm	0.5 ± 0.07	17.5 ± 1.6	-0.96
58 nm	0.6 ± 0.02	28.2 ± 1.5	-0.91

Supplementary References

1. Ulbricht, R., Hendry, E., Shan, J., Heinz, T. F. & Bonn, M. Carrier dynamics in semiconductors studied with time-resolved terahertz spectroscopy. *Rev. Mod. Phys.* **83**, 543–586 (2011).
2. Jänsch, D. *et al.* Ultra-Narrow Low-Bandgap Graphene Nanoribbons from Bromoperylenes-Synthesis and Terahertz-Spectroscopy. *Chem. - Eur. J.* **23**, 4870–4875 (2017).
3. Zheng, W., Zorn, N. F., Bonn, M., Zaumseil, J. & Wang, H. I. Probing Carrier Dynamics in sp^3-Functionalized Single-Walled Carbon Nanotubes with Time-Resolved Terahertz Spectroscopy. *ACS Nano* **16**, 9401–9409 (2022).
4. Jensen, S. A. *et al.* Ultrafast Photoconductivity of Graphene Nanoribbons and Carbon Nanotubes. *Nano Lett.* **13**, 5925–5930 (2013).
5. Kaindl, R. A., Hägele, D., Carnahan, M. A. & Chemla, D. S. Transient terahertz spectroscopy of excitons and unbound carriers in quasi-two-dimensional electron-hole gases. *Phys. Rev. B* **79**, 045320 (2009).
6. Zipfel, J. *et al.* Exciton diffusion in monolayer semiconductors with suppressed disorder. *Phys. Rev. B* **101**, 115430 (2020).
7. Ivanov, I. *et al.* Role of Edge Engineering in Photoconductivity of Graphene Nanoribbons. *J. Am. Chem. Soc.* **139**, 7982–7988 (2017).
8. Hendry, E., Schins, J. M., Candeias, L. P., Siebbeles, L. D. A. & Bonn, M. Efficiency of Exciton and Charge Carrier Photogeneration in a Semiconducting Polymer. *Phys. Rev. Lett.* **92**, 196601 (2004).
9. Wang, F. *et al.* Exciton polarizability in semiconductor nanocrystals. *Nat. Mater.* **5**, 861–864 (2006).
10. Hendry, E. *et al.* Interchain effects in the ultrafast photophysics of a semiconducting polymer: THz time-domain spectroscopy of thin films and isolated chains in solution. *Phys. Rev. B* **71**, 125201 (2005).
