# Supplementary material for: Hot exciton dissociation in graphene nanoribbons
Source: Nat Commun. 2026 Jun 12;17:5260. doi: 10.1038/s41467-026-74158-x (PMC13263337; doi:10.1038/s41467-026-74158-x)
Supplement: Supplementary file 2 — Transparent Peer Review file [file 41467_2026_74158_MOESM2_ESM.pdf]

nature portfolio

Peer Review File

Hot Exciton Dissociation in Graphene Nanoribbons

Corresponding Author: Professor Hai Wang

This file contains all reviewer reports in order by version, followed by all author rebuttals in order by version.

Version 0:

Reviewer comments:

Reviewer #1

(Remarks to the Author)
Referee report on “Hot Exciton Dissociation in Graphene Nanoribbons” by G. Wen, F. Xu, A. Tries, et al.

The manuscript presents a thorough experimental study of the exciton dissociation process in atomically precise graphene nanoribbons (GNRs) using ultrafast optical pump–THz probe spectroscopy. In particular, the authors systematically explore the role of GNR length, pump excitation energy, and carrier effective mass on exciton dissociation, identifying non-equilibrium hot exciton dissociation (HED) as the dominant mechanism for free-carrier generation. The authors also identify a critical Bjerrum length (~20 nm), above which photogenerated electron–hole pairs can effectively dissociate, yielding long-lived free carriers. Interestingly, the authors unambiguously demonstrate that a low effective mass significantly enhances dissociation efficiency, as evidenced by the increased carrier mobility, even in materials with large exciton binding energies.

Overall, the work successfully addresses fundamental questions in excitonic physics in quasi-one-dimensional systems and provides convincing arguments for how free carriers emerge in systems with reduced dimensionality and strong Coulomb interactions, using both a robust experimental technique and controlled, atomically precise one-dimensional systems. These findings certainly provide new directions for the design of excitonic optoelectronic devices. In my opinion, the study is timely and carefully conducted, and could be suitable for publication after some major clarifications regarding methodological details and justification aspects.

1. The information regarding the pump fluence used in the OPTP experiments is not explicitly provided. From my understanding, the manuscript appears to implicitly assume that the experiments are performed within the linear-response regime, where exciton–exciton annihilation and Auger-like processes can be safely neglected. If this is indeed the case, such assumptions should be clearly stated.
Although the photoconductivity is normalized to the absorbed photon density, this normalization alone does not guarantee linearity unless it is explicitly demonstrated that N_abs itself scales linearly with pump fluence over the explored range, and that the THz photoconductivity amplitude also scales linearly with fluence. In the absence of fluence-dependent measurements, it remains unclear whether the reported HED efficiency and the inferred R_B criterion are intrinsic material properties or are partially influenced by density-driven effects.
I encourage the authors to report the pump fluence used in all experiments, along with the corresponding initial photoexcited carrier density. In addition, it would be useful for readers to be provided with experimental evidence demonstrating a linear dependence of the TR-THz signal amplitude on pump fluence.

2. A major conceptual concern arises from the definition and use of the Bjerrum length R_B in the manuscript. The authors employ the 3D (bulk) Bjerrum length,R_B=e^2/4πε_0ε_rk_BT, to rationalize exciton dissociation in GNRs. While this definition is standard for bulk systems with three-dimensional Coulomb interactions, its direct application to quasi-1D systems is not rigorously justified. Strictly speaking, in one-dimensional systems the electrostatic interaction between point charges does not follow a 1/r dependence but instead scales approximately as V(x) ~ -|x-xo|, reflecting reduced dimensionality and field-line confinement (see, e.g., http://doi.org/10.1063/1.3078492). Consequently, the balance between Coulomb attraction and thermal energy—and thus the definition of the Bjerrum length—is inherently dimension dependent. Using a 3D Coulomb potential in a quasi-1D geometry may therefore yield an inappropriate characteristic length scale. This issue is well known in the context of exciton binding energies in reduced-dimensional systems, where 3D Coulomb potentials systematically underestimate binding energies in 2D and 1D materials, while dimension-appropriate interaction models significantly improve agreement with experiments and many-body calculations (see, e.g., Phys. Rev. Lett. 116, 056401 (2016); Phys. Rev. Lett. 113, 076802 (2014); Phys. Rev. B 109, 165425 (2024)). Accordingly, the apparent

agreement between the calculated R_B and experimental crossover lengths may be coincidental or phenomenological. Interpreting R_B as a universal dissociation threshold may therefore not be transferable to other 1D excitonic systems. The authors should either justify the validity of a 3D Coulomb potential for their experimental geometry or discuss how a strictly 1D or quasi-1D interaction would modify R_B and the interpretation of their results.

3. The exciton binding energies E_B are inferred from the excitation-energy dependence of the THz photoconductivity using a model previously introduced by the authors. While this approach is experimentally practical, it relies on several implicit assumptions that are not sufficiently discussed. In particular, the analysis assumes a well-defined energetic threshold separating bound excitons from free carriers and appears to neglect the roles of excited excitonic states, continuum resonances, and nonthermal carrier distributions. In strongly excitonic quasi-1D systems such as GNRs, the dense and nonhydrogenic excitonic spectrum makes it unclear whether the observed crossover can be uniquely associated with a single ground-state E_B. In addition, given the modest length dependence relative to the reported uncertainties, the extracted values are more likely to be regarded as effective rather than strict ground-state binding energies. The manuscript would benefit from clarifying these limitations and the model dependence of the extracted E_B values.

4. The Drude–Smith model is used to analyze the THz photoconductivity, with the back-scattering parameter c attributed to geometrical confinement in one-dimensional structures. This interpretation requires clarification. In quasi-1D GNRs, the extracted c parameter may reflect a combination of end-scattering, edge disorder, defect back-reflection, and partial localization. The manuscript should clarify which microscopic processes are believed to dominate in the present system and why the Drude–Smith description is preferable to alternative models. This point is particularly important because the extracted scattering time and mobility directly support the proposed hot exciton dissociation mechanism.

5. The manuscript reports a charge-carrier mobility of up to ~650 cm^^{2}·V^-1s^-^{1} but does not explicitly provide the expression used to obtain this value. The authors should explicitly provide the expression used to estimate the mobility and clarify whether the reported mobility corresponds to a short-range or long-range diffusive mobility.

6. While the manuscript discusses exciton formation and free-carrier lifetimes on picosecond to nanosecond timescales, radiative exciton recombination is not quantitatively addressed. In 1D systems, radiative decay can be a significant competing channel that influences apparent carrier lifetimes and dissociation efficiencies. The authors might consider to discuss expected radiative lifetimes based on literature values or oscillator-strength considerations and clarify whether radiative recombination is assumed to be negligible within the experimental time window.

Overall, this is a very good and timely paper with relevant results, but I believe it would benefit from addressing the questions raised above.

Reviewer #2

(Remarks to the Author)
The authors report conductivity measurements of graphene nanoribbons (GNRs) to find a critical length beyond which exciton dissociation dominates carrier generation. Experiments are based on ultrafast Terahertz conductivity measurements of different types of GNRs, grown in solution with length control. The authors find that for the 58 nm long GNRs, 30 % of the carriers remain unbound after 1 ps and up to 1 ns. The authors discuss differences in the length-dependent free carrier lifetimes using the Bjerrum length that is calculated to be ~20 nm.
While the experimental data are generally of high quality and several competing models can be excluded, the physical interpretation based on the Bjerrum length remains overly simplistic. In particular, the model does not adequately capture the dynamics in a strictly
one-dimensional material. As a result, several key conceptual and quantitative issues require clarification before the work can be considered for publication.

• The authors discuss the length-dependent electronic properties only in the introduction and use a reference (Talirz et al.) to conclude that GNRs electronic band gaps approach the value of infinite GNRs within 50 meV for a length beyond 8 nm. However, discussion on the contribution of the effective mass falls way too short. The critical length does sensitively depend on the effective mass, especially for the case of the first GNR with a mass as low as me/m0 = 0.08, a value the authors take from Ref. 43. This needs to be discussed in detail for the three different lengths of the investigated GNRs.

• An critical question related to the 1D confinement of charge carriers in GNRs is not addressed in this model. What is the expected temporal evolution of separated charge carriers in this 1D system? Scattering at ends together with the suggested long lifetime suggests that separated electrons and holes should collide multiple times due to scattering at the GNR ends. This should be addressed theoretically for different GNR lengths.

• Related to this, the Bjerrum-length concept has be reconsidered. In the current discussion, important points relevant to a low-dimensional material are not discussed. This includes
• role of dimensionality in modifying the coulomb interaction
• local and anisotropic dielectric screening near the GNR
• the fact that carriers are initially 'hot', whereas R_B is defined using k_B T.

Minor points:

• In Fig. 2d: only very few points with different pump energies are measured for the 6 nm GNRs. Why does this differ from the other GNRs and what why is the error bar (not visible for 6 nm GNRs) so much smaller? Scaling factors should be simplified by choosing one (unscaled) reference curve. Also, mixing multiplication and division factors adds unnecessary confusion.
• In Fig 4d: Solid lines are described as single exponential functions. This needs further details. What exact function is used to fit the delay time-dependent exciton fraction?

Reviewer #3

(Remarks to the Author)
The manuscript presents a clear storyline, and most of the claims are well supported by theory. The authors investigate a strong ~1 eV tightly bound exciton in ~1 nm-wide GNRs, which enables hot exciton dissociation (HED) to remain stable in longer ribbons (lengths > 2 × 20 nm) compared to other low-dimensional organic material systems where the exciton binding energy (E_B) is typically below 1 eV.
They perform ultrafast terahertz (THz) conductivity measurements on:
• 6, 11, and 58 nm AHM-GNRs with ~0.08 m_0 carrier effective mass,
• 70 nm CMGNR with ~0.8 m_0 effective mass,
• 43 nm HTGNR with similar effective mass to AHM-GNR.
These measurements systematically demonstrate that for GNRs with high mobility (i.e., low effective mass), HED can exist and contribute up to ~30% of the total carrier in the ribbon.
The study of hot excitons using THz photoconductivity, decomposed into free-carrier and excitonic contributions via a Drude–Smith plus low-frequency excitonic model, and the extraction of exciton fractions versus time for different lengths is intuitive and well executed.
This work extends the previous story of “strong exciton” in GNRs (Nano Lett. 20, 2993–3002 (2020) from the same group) from static binding energy and for different GNR formation to length-dependent hot carrier dissociation. The experimentally grounded Bjerrum-length framework in a strongly excitonic 1D system is also valuable. This is likely to interest not only the GNR community but also researchers in broader excitonic material platforms. Most of the claims and conclusions appear justified.
Questions for the Authors
1. Width variations and localization
Given that the width of the GNRs can vary between 1 and 1.7 nm, could atomic-scale variations localize excitons? These fluctuations could behave as 1D quantum wells. If so:
Could they contribute to the reactive negative imaginary conductance?
How can their contribution be distinguished from that of free excitons?
2. Applicability to device platforms
Since the suspension GNR system lacks a substrate and electrical contacts, how does this translate to practical applications such as photodetectors, solar cells, and phototransistors? These devices must be substrate-supported and electrically (or capacitively) contacted, and these factors could significantly modify mobility, screening, and heat dissipation.
3. Regarding Fig. S1
Why is the photoconductivity at 0.5 ps for the 11 nm AHM-GNR approximately twice lower than that for the 6 nm and 58 nm samples?
Suggestions
• A TEM image of the GNRs would be a strong experimental confirmation of uniformity for the synthesized structures.
• Future studies (not needed for this paper) could investigate substrate effects on hot exciton dissociation, since mobility, charge screening, and heat dissipation would directly impact exciton stability (possibly reducing the stability) and therefore device performance. Understanding this dependence could lead to device configurations that are better match for photodetector, solar cell, and phototransistor applications.
Overall Assessment
The methodology is clearly described, the measurements appear sound, and the work meets the standards of the field. The provided methods and references should enable experimental reproducibility.

Version 1:

Reviewer comments:

Reviewer #1

(Remarks to the Author)
The authors have provided thorough responses, offering reasonable arguments as well as complementary experimental measurements (where appropriate) to address the points raised by the three reviewers. In particular, I am satisfied with how the authors have addressed my main concern regarding the Bjerrum length approximation. In the revised manuscript, the validity of this approximation is now clearly stated.

Although the inclusion of Ref. 11 is essential to support their response, I believe that the key statement presented in the replies to Referee #1 and Referee #2 should also be incorporated into the revised manuscript, i.e.,

"In our system, the GNRs are narrow but embedded in a liquid solvent with a comparable or higher dielectric constant. Under these conditions, the short-range excitonic physics is governed by the quasi-1D, strongly bound regime, whereas at larger separations the Coulomb interaction is expected to be dominated by solvent screening and can be reasonably described by a 3D-screened potential [Phys. Rev. B 2024, 109, 165425]."

Including this statement would further strengthen the manuscript. With this minor revision, I consider the paper suitable for publication in Nature Communications.

Reviewer #3

(Remarks to the Author)
The authors have addressed the concerns and suggestions I raised in the previous round of review. The revised manuscript is now clear, with well-supported physical interpretations and improved clarity regarding the practical applications. I have no further questions and recommend this manuscript for publication.

Open Access This Peer Review File is licensed under a Creative Commons Attribution 4.0 International License, which permits use, sharing, adaptation, distribution and reproduction in any medium or format, as long as you give appropriate credit to the original author(s) and the source, provide a link to the Creative Commons license, and indicate if changes were made.
In cases where reviewers are anonymous, credit should be given to 'Anonymous Referee' and the source.
The images or other third party material in this Peer Review File are included in the article's Creative Commons license, unless indicated otherwise in a credit line to the material. If material is not included in the article's Creative Commons license and your intended use is not permitted by statutory regulation or exceeds the permitted use, you will need to obtain permission directly from the copyright holder.
To view a copy of this license, visit https://creativecommons.org/licenses/by/4.0/

Point-by-point response to referees' comments

Manuscript ID: NCOMMS-25-098713
Manuscript Type: Article
Title: Hot Exciton Dissociation in Graphene Nanoribbons
Author(s): Guanzhao Wen, Fugui Xu, Alexander Tries, Wenhao Zheng, Lucia Di Virgilio, Shuai Fu, Xinyu Chen, Lin Yang, Zijie Xiao, Mathias Kläui, Silvio Osella, Ji Ma, Xu Wang, Xinliang Feng, Yiyong Mai, Mischa Bonn, and Hai I. Wang.

We thank the reviewers for their constructive and insightful comments, which have substantially improved the clarity and robustness of the manuscript. In response, we have (i) added fluence-dependent OPTP measurements demonstrating that all data were taken in the linear-response regime; (ii) clarified the conceptual role of the Bjerrum length in quasi-1D GNRs, emphasizing its interpretation as an environment-controlled, long-range scale rather than a universal 1D dissociation threshold; (iii) explicitly rephrased all exciton binding energies as "apparent/effective" values and discussed the model dependence and spectral complexity; (iv) refined our analysis of the THz photoconductivity using the Drude–Smith model, clearly distinguishing geometric and physical back-scattering and specifying that the reported mobility is a short-range, early-time quantity; and (v) added discussion and literature context on radiative lifetimes, effective mass, the temporal evolution of separated carriers in finite-length GNRs, and the possible influence of the dielectric/substrate environment on hot-exciton dissociation and device-relevant performance. We have revised the main text and Supporting Information accordingly to reflect these changes, temper claims where necessary, and improve readability while preserving the work's central conclusions.

A point-by-point response to the reviewers' comments and suggestions can be found below. Answers in the rebuttal are marked in blue; changes to the manuscript and Supporting Information are marked in red.

REVIEWER COMMENTS

Reviewer #1 (Remarks to the Author):

Referee report on “Hot Exciton Dissociation in Graphene Nanoribbons” by G. Wen, F. Xu, A. Tries, et al.

“The manuscript presents a thorough experimental study of the exciton dissociation process in atomically precise graphene nanoribbons (GNRs) using ultrafast optical pump–THz probe spectroscopy. In particular, the authors systematically explore the role of GNR length, pump excitation energy, and carrier effective mass on exciton dissociation, identifying non-equilibrium hot exciton dissociation (HED) as the dominant mechanism for free-carrier generation. The authors also identify a critical Bjerrum length (~20 nm), above which photogenerated electron–hole pairs can effectively dissociate, yielding long-lived free carriers. Interestingly, the authors unambiguously demonstrate that a low effective mass significantly enhances dissociation efficiency, as evidenced by the increased carrier mobility, even in materials with large exciton binding energies.

Overall, the work successfully addresses fundamental questions in excitonic physics in quasi-one-dimensional systems and provides convincing arguments for how free carriers emerge in systems with reduced dimensionality and strong Coulomb interactions, using both a robust experimental technique and controlled, atomically precise one-dimensional systems. These findings certainly provide new directions for the design of excitonic optoelectronic devices. In my opinion, the study is timely and carefully conducted and could be suitable for publication after some major clarifications regarding methodological details and justification aspects.”

and…

“Overall, this is a very good and timely paper with relevant results, but I believe it would benefit from addressing the questions raised above.”

Response: We would like to thank the reviewer for the positive assessment of our manuscript. In the following, we will provide point-by-point responses to the comments raised by the reviewer.

1. The information regarding the pump fluence used in the OPTP experiments is not explicitly provided. From my understanding, the manuscript appears to implicitly assume that the experiments are performed within the linear-response regime, where exciton–exciton annihilation and Auger-like processes can be safely neglected. If this is indeed the case, such assumptions should be clearly stated.

Although the photoconductivity is normalized to the absorbed photon density, this normalization alone does not guarantee linearity unless it is explicitly demonstrated that N_{abs} itself scales linearly with pump fluence over the explored range, and that the THz photoconductivity amplitude also scales linearly with fluence. In the absence of fluence-dependent measurements, it remains unclear whether the reported HED efficiency and the inferred R_B criterion are intrinsic material properties or are partially influenced by density-driven effects.

I encourage the authors to report the pump fluence used in all experiments, along with the corresponding initial photoexcited carrier density. In addition, it would be useful for readers to be provided with experimental evidence demonstrating a linear dependence of the TR-THz signal amplitude on pump fluence.

Response: We understand the reviewer's concern about the pump fluence conditions and the linearity of the OPTP response. To verify that the measurements were performed in the linear-response regime, we measured the pump-fluence dependence of the THz photoconductivity measurements ($-\Delta E/E$). As shown in the attached figure and the revised Supporting Information Figures S1, S2 and S7, the peak amplitudes scale linearly with the absorbed photon density over the fluence range explored in this work for the representative samples and excitation photon energies. Furthermore, the normalized dynamics overlap with all fluences for a given pump wavelength; see examples provided in Figures S2 and S7. This linear dependence of the amplitude and independence of the dynamics indicate that the reported OPTP response is proportional to the initial photoexcited carrier density, and that higher-order density-dependent processes such as exciton-exciton annihilation or Auger-like recombination are negligible under our experimental conditions.

Action: In the revised manuscript (Lines 199-203, Pages 8-9), we state that "*We note that within the range of pump fluences used in our studies, for a given pump photon energy hv, all photoconductivity dynamics scale with the absorbed photon density.*

Specifically, the peak photoconductivity values scale linearly with N_{abs} (see Figure S1), while the normalized dynamics overlap with one another (see Figure S2)." In the revised Supporting Information, we have added the pump fluence-dependent THz photoconductivity results, see Figure S1, Page S4; Figure S2, Page S5; and Figure S7, Page S8.

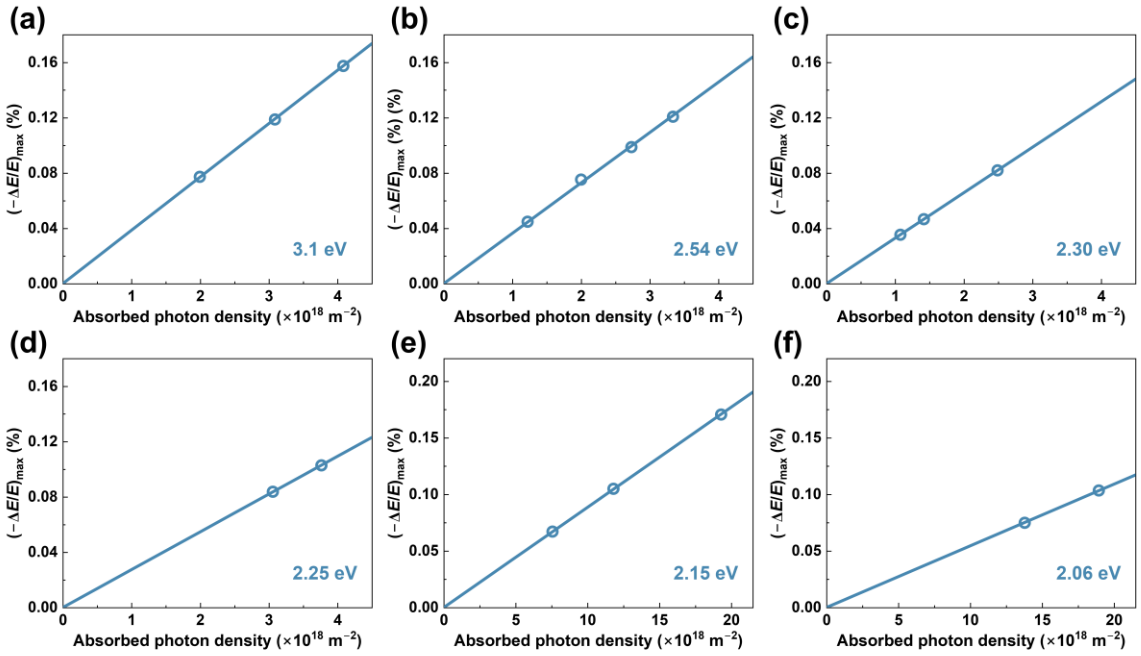

Figure S1. Representative pump-fluence dependence of the peak THz photoconductivity. The peak THz signal $(-\Delta E/E)_{max}$ is plotted as a function of the absorbed photon density for (a-f) the 58 nm AHM-GNR dispersion at various excitation photon energies ranging from 2.06 eV to 3.1 eV. The open circles represent experimental data, and the solid lines are the corresponding linear fits.

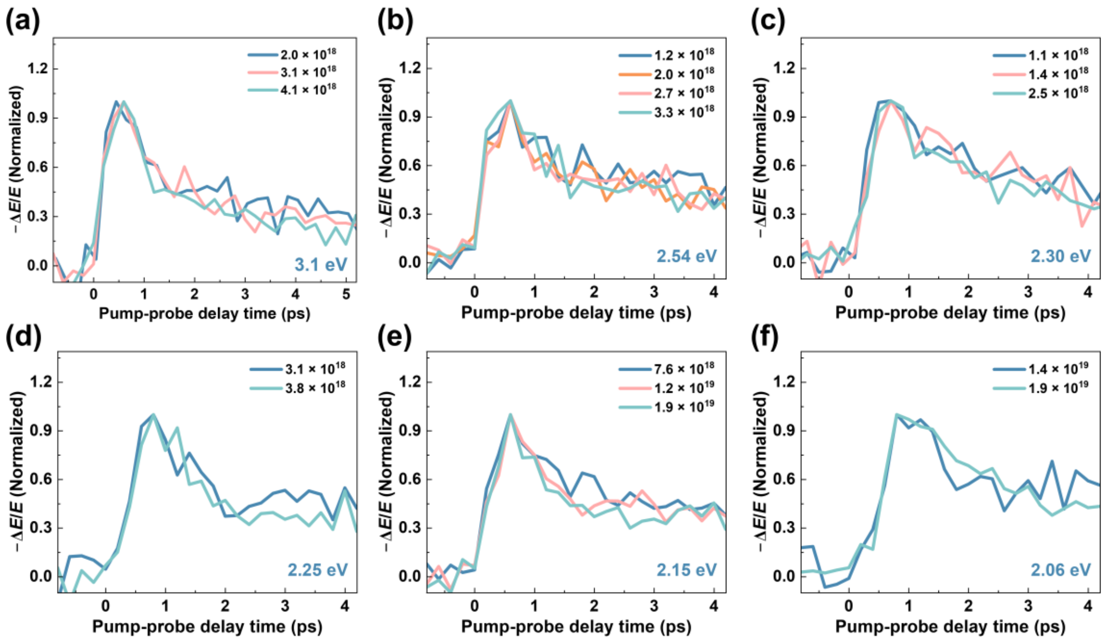

Figure S2. Representative pump-fluence dependence of the time-resolved THz photoconductivity dynamics. The normalized time-resolved THz photoconductivity is plotted as a function of the absorbed photon density (N_{abs}, m^{-2}) for (a-f) the 58 nm AHM-GNR dispersion at various excitation photon energies ranging from 2.06 eV to 3.1 eV.

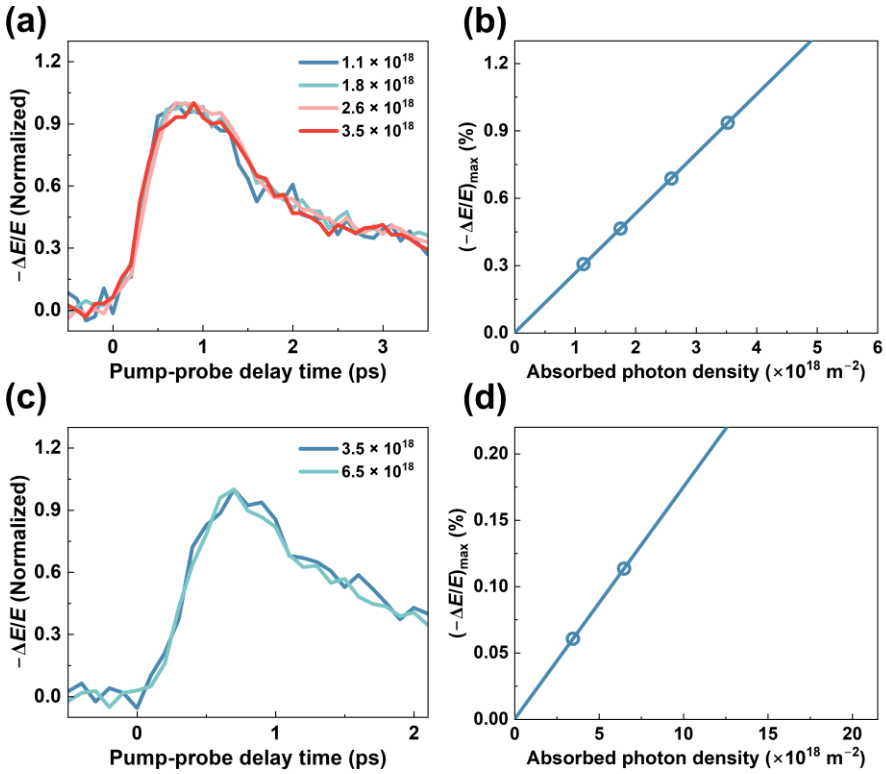

Figure S7. Fluence-dependent photoconductivity dynamics. Panels (a) and (c) show the normalized time-resolved THz photoconductivity, while panels (b) and (d) present the corresponding peak photoconductivity $(-\Delta E/E)_{max}$ for (a, b) the HTGNR dispersion and (c, d) the cMGNR dispersion under 3.1 eV excitation. Open circles represent experimental data, and solid lines in (b) and (d) are linear fits.

2. A major conceptual concern arises from the definition and use of the Bjerrum length R_B in the manuscript. The authors employ the 3D (bulk) Bjerrum length, $R_B = e^2/4\pi\varepsilon_0\varepsilon_r k_B T$, to rationalize exciton dissociation in GNRs. While this definition is standard for bulk systems with three-dimensional Coulomb interactions, its direct application to quasi-1D systems is not rigorously justified. Strictly speaking, in one-dimensional systems the electrostatic interaction between point charges does not follow a $1/r$ dependence but instead scales approximately as $V(x) \sim -|x-x_o|$, reflecting reduced dimensionality and field-line confinement (see, e.g., http://doi.org/10.1063/1.3078492). Consequently, the balance between Coulomb attraction and thermal energy—and thus the definition of the Bjerrum length—is inherently dimension dependent. Using a 3D Coulomb potential in a quasi-1D geometry may therefore yield an inappropriate characteristic length scale. This issue is well known in the context of exciton binding energies in reduced-dimensional systems, where 3D Coulomb potentials systematically underestimate binding energies in 2D and 1D materials, while dimension-appropriate interaction models significantly improve agreement with experiments and many-body calculations (see, e.g., Phys. Rev. Lett. 116, 056401 (2016); Phys. Rev. Lett. 113, 076802 (2014); Phys. Rev. B 109, 165425 (2024)). Accordingly, the apparent agreement between the calculated R_B and experimental crossover lengths may be coincidental or phenomenological. Interpreting R_B as a universal dissociation threshold may therefore not be transferable to other 1D excitonic systems. The authors should either justify the validity of a 3D Coulomb potential for their experimental geometry or discuss how a strictly 1D or quasi-1D interaction would modify R_B and the interpretation of their results.

Response: We thank the reviewer for this important comment on the dimensionality of the Coulomb interaction and the use of the Bjerrum length (R_B) in quasi-1D GNRs. We fully agree that a strictly 1D electrostatic problem does not follow a $1/r$ potential, and that our use of the 3D R_B constitutes an approximation. In reduced-dimensional systems, however, theory and experiment show that environmental screening can restore an effectively 3D-like $1/r$ interaction at distances much larger than the size of excitons, while short-range interactions remain strongly enhanced [JETP Lett. 1979, 29, 658−660; Phys. Rev. Lett. 2014, 113, 076802; Nat. Commun. 2017, 8, 15251]. and quasi-1D in case of GNRs [Phys. Rev. B 2024, 109, 165425].

In our system, the GNRs are narrow but embedded in a liquid solvent with a comparable or higher dielectric constant. Under these conditions, the short-range excitonic physics

is governed by the quasi-1D, strongly bound regime, whereas at larger separations the Coulomb interaction is expected to be dominated by solvent screening and can be reasonably described by a 3D-screened potential [Phys. Rev. B 2024, 109, 165425]. The hot exciton dissociation process we discuss is therefore sequential: immediately after excitation, the "hot" electron-hole pairs first overcome the strong short-range confinement; the decisive criterion for generating long-lived free carriers is then whether they can separate further, before cooling, to a distance at which the remaining, solvent-screened Coulomb attraction drops below $k_B T$. This latter, long-range step that we describe using the conventional Bjerrum length $R_B = e^2/(4\pi\varepsilon_0\varepsilon_r k_B T)$. This macroscopic value reflects the distance at which the thermal energy matches the Coulomb potential between carriers, which we interpret as the projected separation along the ribbon at which the 3D-screened interaction equals the thermal energy.

To sum up, while short-range exciton physics is governed by the quasi-1D nature of the ribbon, the long-range criterion relevant to free-carrier escape probability is set by the surrounding 3D solvent environment. The consistency between the experimentally observed threshold (between 11 and 58 nm) and the estimated $2R_B$ supports the physical validity of this kinetic escape model.

Action: We have revised the manuscript to clearly state that the use of the 3D Bjerrum length is an environment-controlled, long-range approximation (see Lines 299-311, Page 13).

"*…Instead, we attribute the length-dependent generation of free carriers in GNRs to a kinetic process governed by the interplay between ribbon length and the effective spatial range of Coulomb attraction. The Bjerrum length $R_B = e^2/(4\pi\varepsilon_0\varepsilon_r k_B T)$,[56–58] reflects the distance where thermal energy matches the Coulomb potential between carriers, where ε_r, k_B, and T represent the relative permittivity of the solvent, the Boltzmann constant, and the temperature, respectively. We note that charge carriers in GNRs are confined to a 1D geometry, resulting in strong excitonic effects.[11] As such, in*

the early time scale following optical excitation, excitation above the bandgap generates transient "hot" free carriers, and only those with sufficiently high kinetic energy can effectively overcome the strong short-range 1D Coulomb potential for charge separation: greater photon energies yield excess kinetic energy, allowing electrons and holes to separate more effectively. Following this "initial" dissociation process, however, the decisive criterion for generating long-lived free carriers is then whether they can separate further, before cooling, to a distance where the remaining, solvent-screened Coulomb attraction drops below k_BT. As this critical length scale is much longer than the exciton length scale (on the order of 1 nm), the long-range interaction is effectively mediated by the surrounding 3D liquid environment. Consequently, we consider a fully solvent-screened 3D Coulomb potential, rather than a strictly 1D form, for estimating R_B."

3. The exciton binding energies E_B are inferred from the excitation-energy dependence of the THz photoconductivity using a model previously introduced by the authors. While this approach is experimentally practical, it relies on several implicit assumptions that are not sufficiently discussed. In particular, the analysis assumes a well-defined energetic threshold separating bound excitons from free carriers and appears to neglect the roles of excited excitonic states, continuum resonances, and nonthermal carrier distributions. In strongly excitonic quasi-1D systems such as GNRs, the dense and nonhydrogenic excitonic spectrum makes it unclear whether the observed crossover can be uniquely associated with a single ground-state E_B. In addition, given the modest length dependence relative to the reported uncertainties, the extracted values are more likely to be regarded as effective rather than strict ground-state binding energies. The manuscript would benefit from clarifying these limitations and the model dependence of the extracted E_B values.

Response: We agree that extracting an exciton binding energy from the excitation-energy dependence of the THz photoconductivity relies on several assumptions that require clarification. We agree that the inferred E_B is indeed an apparent one that is inferred from the energy difference between the optical bandgap and the transition energy from the photoconductivity onset to its saturation by increasing *hv*. Our

approach is consistent with earlier conductivity-based studies of excitonic systems, most notably in conjugated polymers, where the inflection or onset of the photoconductivity excitation profile was used to estimate an effective exciton binding energy while explicitly acknowledging contributions from excited excitonic states and near-continuum resonances [e.g., PNAS 2001, 98, 13496-13500]. The extracted energy scale is understood as phenomenological and model-dependent, rather than as a strict ground-state quantity.

Regarding the reviewer's specific concerns, excited excitonic states, continuum resonances, nonthermal carrier distributions, and the dense and nonhydrogenic excitonic spectrum can, in principle, broaden the crossover region by enabling partial or state-dependent ionization pathways. In particular, the intra-excitonic absorption can, in principle, contribute to the apparent THz absorption, to some extent, to "blur" the transition from the exciton to the electronic band continuum. Furthermore, given the probe time resolution of OPTP is ~200 fs at best, the excitation will definitely evolve from a sharp, nonthermal initial excitation following the pump. However, they do not invalidate the use of the crossover itself as an experimentally accessible energy scale for free-carrier generation. Here, to estimate the potential role of all these effects in definitively defining the threshold, we took the first derivative of the fitted excitation-energy-dependent photoconductivity (as an example for 58 nm), as shown in the attached figure. This result reveals that the transition from the insulating regime to the saturated photoconductivity plateau is characterized by a relatively narrow peak (FWHM $\approx$ 0.14 eV), rather than a broad, structureless feature. This sharpness confirms that, despite the underlying spectral complexity, the mobile free-carrier generation occurs over a well-defined energy range.

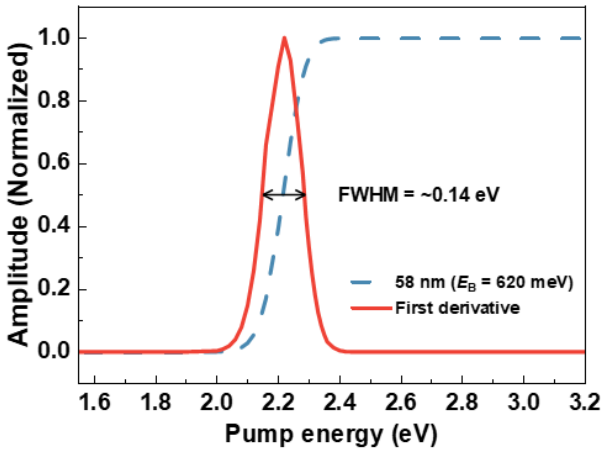

Analysis of the exciton dissociation threshold in 58 nm AHM-GNRs. The dashed blue curve represents the fitting curve of the pump-energy-dependent photoconductivity for the 58 nm AHM-GNR (corresponding to the regression analysis shown in Figure 2d in the main text). The solid red curve depicts the first derivative of this fitted curve. The derivative reveals a distinct, relatively narrow peak centered at the threshold energy, with a full width at half maximum (FWHM) of ~0.14 eV.

Action: We have revised the manuscript to explicitly state that the reported E_B values represent an apparent exciton binding energy and should not be interpreted as strict ground-state exciton binding energies in GNRs, see Lines 249-252, Page 10.

"*…The fits, shown as dashed lines in Figure 2d, yield E_B of 730 ± 65, 700 ± 50, and 620 ± 55 meV for AHM-GNRs with lengths of 6, 11, and 58 nm, respectively. Such an inverse scaling between E_B and l can be qualitatively understood as a consequence of the increased delocalization of electron-hole wavefunctions and the reduced quantum confinement that occurs with increasing ribbon length.[53] Given the potentially dense and nonhydrogenic excitonic spectrum inherent to GNRs, the optical transition to the continuum can be broadened by higher-lying excited states and resonances. Hence, the E_B values extracted here represent effective exciton binding energies rather than strict ground-state quantities. Moreover, we note that the E_B for the longest ribbons is found to be still large, over 600 meV, demonstrating the general exciton-dominated optical properties in narrow, atomically precise GNRs…*"

4. The Drude–Smith model is used to analyze the THz photoconductivity, with the back-scattering parameter *c* attributed to geometrical confinement in one-dimensional structures. This interpretation requires clarification. In quasi-1D GNRs, the extracted *c* parameter may reflect a combination of end-scattering, edge disorder, defect back-reflection, and partial localization. The manuscript should clarify which microscopic processes are believed to dominate in the present system and why the Drude–Smith description is preferable to alternative models. This point is particularly important because the extracted scattering time and mobility directly support the proposed hot exciton dissociation mechanism.

Response: We thank the reviewer for this insightful comment about the physical interpretation of the Drude-Smith (DS) parameter. In our solution experiments, the GNRs form a disordered ensemble with an isotropic angular distribution within the three-dimensional solvent. Under linearly polarized THz excitation, the macroscopic conductivity represents an average of the carrier motion (following Drude response) projected onto the THz field axis. Theoretical modeling of such 1D conductors with random 3D orientation predicts a geometric back-scattering parameter of approximately $c \sim -0.79$ (or $-\pi/4$), arising purely from this geometrical projection of Drude conductivity rather than intrinsic defects or scattering to e.g., their ends [Nano Lett. 2013, 13, 5925−5930]. As such, the extracted large *c* values (−0.95, −0.96, and −0.91) indicate that a significant contribution may come from the 1D confinement within a 3D disordered ensemble. However, as the absolute value of the inferred *c* is still greater than $-\pi/4$, the extraction of scattering contributions should be considered. In this line, a previous study by Ivanov et al. showed a universal carrier back-scattering effect in several GNRs due to the conformational flexibility, leading to the formation of structural bends and links along the ribbon backbone, with an average momentum-scattering time of ~30 fs [Nano Lett. 2013, 13, 5925−5930; J. Am. Chem. Soc. 2017, 139, 7982−7988]. This value is close to what we inferred for the longest ribbons of ~28 fs, but higher than that of short ribbons of 6 and 11 nm. This result may indicate that

scattering, e.g., to the ends of GNRs, may play a role in the short ribbons below 11 nm. Consequently, the Drude-Smith model provides a reasonable, phenomenological description of this specific frequency dependence, characterized by a suppressed *dc* conductivity, which arises from the combined effects of geometric disorder, GNR ends, and structural flexibility, a regime not accessible to standard Drude or Lorentzian models. Moreover, while the extracted momentum-scattering time may exhibit some inherent model dependence and is constrained by the finite frequency bandwidth of our measured spectrometer, the exceptionally high carrier mobility originates fundamentally from the small effective mass of the GNRs. Thus, our core mechanistic conclusions regarding the hot-exciton dissociation process and its dependence on carrier mobility remain valid.

Action: Following this suggestion and our reply above, we added the above information to the revised manuscript in Lines 219-228, Page 9.

"*…In contrast, momentum scattering from defects, local structural deformations, and phonons within the ribbons dominates conduction in longer GNRs.*[34,51] *In this work, we applied the Drude-Smith model to capture the complex interplay of scattering mechanisms. Because the GNRs form a disordered ensemble in solution, orientational averaging of the 1D carrier motion inherently yields a geometric back-scattering footprint of c ≈ −0.79.*[41] *The extracted c values (various −0.91 to −0.96) exceed this purely geometric limit, reflecting additional physical back-scattering that suppresses long-range transport. This excess back-scattering may arise from conformational bends or defects in extended ribbons, whereas finite-length boundary (end) scattering is expected to become more important in shorter ribbons.*[41] *Accordingly, the Drude-Smith model serves as a suitable phenomenological tool to account for the suppressed dc conductivity originating from both geometric projection and intra-ribbon scatterings.*"

5. The manuscript reports a charge-carrier mobility of up to ~650 $cm^2 \cdot V^{-1} s^{-1}$ but does not explicitly provide the expression used to obtain this value. The authors should explicitly provide the expression used to estimate the mobility and clarify whether the reported mobility corresponds to a short-range or long-range diffusive mobility.

<u>Response:</u> We thank the reviewer for pointing this out. The charge-carrier mobility (~650 $cm^2 \cdot V^{-1} s^{-1}$) reported in the manuscript is obtained via assuming diffusive transport along ribbon, following the equation as: $\mu = e\tau / m^*$, where e is the elementary charge, τ is the effective momentum scattering time obtained from the Drude-Smith fit (~28 fs for AHM-GNR with averaged length of 58 nm), and m^* represents the reduced effective carrier mass of AHM-GNRs (~0.08 m_0). The mobility extracted in this way corresponds to an intrinsic, local short-range mobility, which reflects the initial carrier response following photoexcitation before carriers experience multiple scattering events.

<u>Action:</u> We have clarified this point in the revised manuscript and explicitly provided the expression used to estimate the mobility, see Lines 323-328, Page 13.

"…*Combined with the measured scattering time ($\tau \approx 28$ fs), we estimate a short-range charge carrier mobility (μ) in our GNRs to be up to ~ 650 $cm^2 \cdot V^{-1} \cdot s^{-1}$ using the expression as $\mu = e \cdot \tau / m^*$, where e is the elementary charge. This mobility value is inferred from THz photoconductivity and thus represents short-range, high-frequency charge transport at the nanometer length scale, rather than macroscopic device mobilities.*"

6. While the manuscript discusses exciton formation and free-carrier lifetimes on picosecond to nanosecond timescales, radiative exciton recombination is not quantitatively addressed. In 1D systems, radiative decay can be a significant competing channel that influences apparent carrier lifetimes and dissociation efficiencies. The authors might consider discussing expected radiative lifetimes based on literature values or oscillator-strength considerations and clarify whether radiative recombination is assumed to be negligible within the experimental time window.

Response: We agree that in one-dimensional systems, radiative decay can be sufficiently fast to limit the carrier lifetime and exciton dissociation efficiencies. The time-resolved photoluminescence (TRPL) measurement on the same AHM-GNR dispersion (with 11 nm) has been previously investigated [Nano Lett. 2020, 20, 2993-3002], which shows the exciton PL lifetime up to ~8 ns. The exciton dissociation and free-carrier lifetime discussed in the present work occur on much shorter time scales. As a result, we believe that radiative decay would not compete with the early-, sub-ps -time processes discussed in our work here.

Action: We have clarified this separation of time scales in the revised manuscript, see Lines 280-287, Page 12.

"*…Our results show that, for short ribbons (6 and 11 nm), excitons rapidly become the dominant species within 10 ps after excitation. In contrast, long ribbons (58 nm) retain a significant population of long-lived free carriers, with about 30% of the carriers remaining unbound even after 10 ps. Across all samples, this composition stabilizes on the picosecond timescale and remains nearly constant up to 1 nanosecond (ns). We note that, within the sub-ns window, radiative recombination can be a significant competing channel in many one-dimensional nanostructures. Previous time-resolved photoluminescence measurement on the same 11 nm AHM-GNR dispersions reported an exciton radiative timescale up to ~8 ns.[11] Given that this radiative decay timescale is substantially longer than our maximum observation window (~1 ns) and the sub-ps exciton dissociation processes, we expect that radiative losses play a relatively minor role in the early-time carrier and exciton dynamics discussed here.*"

Reviewer #2 (Remarks to the Author):

The authors report conductivity measurements of graphene nanoribbons (GNRs) to find a critical length beyond which exciton dissociation dominates carrier generation. Experiments are based on ultrafast Terahertz conductivity measurements of different types of GNRs, grown in solution with length control. The authors find that for the 58 nm long GNRs, 30 % of the carriers remain unbound after 1 ps and up to 1 ns. The authors discuss differences in the length-dependent free carrier lifetimes using the Bjerrum length that is calculated to be ~20 nm.

While the experimental data are generally of high quality and several competing models can be excluded, the physical interpretation based on the Bjerrum length remains overly simplistic. In particular, the model does not adequately capture the dynamics in a strictly one-dimensional material. As a result, several key conceptual and quantitative issues require clarification before the work can be considered for publication.

Response: We thank the reviewer for the careful evaluation of our manuscript and for the constructive comments, which helped to improve the clarity of the manuscript. We have carefully considered each point and revised the manuscript accordingly. Detailed responses to the individual comments are provided below.

- The authors discuss the length-dependent electronic properties only in the introduction and use a reference (Talirz et al.) to conclude that GNRs electronic band gaps approach the value of infinite GNRs within 50 meV for a length beyond 8 nm. However, discussion on the contribution of the effective mass falls way too short. The critical length does sensitively depend on the effective mass, especially for the case of the first GNR with a mass as low as m_e/m_0 = 0.08, a value the authors take from Ref. 43. This needs to be discussed in detail about the three different lengths of the investigated GNRs.

Response: We thank the reviewer for this comment. In GNRs, the carrier effective mass is critically determined by the transverse confinement (ribbon width) and the edge geometry, which dictate the bandedge dispersion relations. [Phys. Rev. Lett. 2006, 97, 216803; Phys. Rev. B 2014, 89, 245407] When the ribbon length goes beyond e.g. 5 nm, it has been well documented that the size of the electronic bandgaps reaches a saturation value [ChemPhysChem 2019, 20, 2348–2353; J. Phys. Chem. C 2022, 126, 1946–1957; Nat. Commun. 2015, 6, 10177]. Therefore, for the three GNR lengths studied here, it is reasonable to speculate that the length does not strongly affect the

effective mass for our samples, as all lengths exceed the saturation threshold. Accordingly, the effective mass value taken from Ref. 43, which pertains to ribbons with the same edge structure, is adopted as a reasonable estimate for all samples.

Action: We have clarified this corresponding description in the revised manuscript, see Lines 317-323, Page 13.

"*...Efficient HED in GNRs relies on excess energy and high charge carrier mobility, particularly of hot charge carriers, to enable separation before rapid exciton formation. Indeed, the high charge carrier mobility in GNRs could balance the large excitonic effect, leading to efficient free carrier generation by HED. In estimating this mobility, we adopt the reported effective mass ($m^* \approx 0.08\ m_0$),[43] as a common value for the three GNRs studied here. It is well established that in GNRs, the carrier effective mass is primarily determined by ribbon width and edge geometry, which dictate the band-edge dispersion.[61,62] When increasing the length of GNRs, bandgap saturation occurs once the length exceeds ~5-8 nm (depending on the ribbon structure).[37–39] Since all three GNR lengths exhibit optical bandgap saturation, we do not expect the effective mass to show a significant length dependence.*"

• A critical question related to the 1D confinement of charge carriers in GNRs is not addressed in this model. What is the expected temporal evolution of separated charge carriers in this 1D system? Scattering at ends together with the suggested long lifetime suggests that separated electrons and holes should collide multiple times due to scattering at the GNR ends. This should be addressed theoretically for different GNR lengths.

Response: We thank the reviewer for raising this interesting question on the temporal evolution of separated charges in GNR system and the role of end scattering. As a representative example, we provide an estimate of the diffuse property for the longest AHM-GNR sample (~58 nm) and a physical discussion to clarify why long-lived

photoconductivity is still compatible with finite ribbon length. We extract a momentum scattering time (τ_{DS}) as ~28 fs for AHM-GNR via fitting the frequency-dependent photoconductivity by Drude-Smith model. Combining the effective mass (m^*) of ~0.08 m_0, this corresponds to an intrinsic mobility (μ) as ~610 $cm^2V^{-1}s^{-1}$ ($\mu = e\tau/m^*$) in the early time scale before carrier relaxation. Hence, via the Einstein relation, this yields a diffusion coefficient (D) of ~16 cm^2s^{-1} at room temperature. For a carrier lifetime (τ) on the order of ~1 ns, the ideally corresponding diffusion length ($L = (2D\tau)^{1/2}$) is ~1-2 μm, which is indeed more than an order of magnitude larger than the ribbon length. This implies that, in a purely diffusive picture of bare charge carriers, electrons and holes traverse the ribbon multiple times and undergo frequent scattering at the ends.

The apparent inconsistency between the long-lived charge separation and the high carrier mobility may point to a more complex transport mechanism, e.g. involving e.g. polaron formation following charge separation and relaxation on a ps time scale. Crucially, as suggested by the strong electron-phonon coupling inherent to soft organic nanostructures, these separated carriers are expected to relax into polaron states. [Adv. Mater. 2010, 22, 3893–3898; Chem. Rev. 2007, 107, 926–952; ACS Nano 2025, 19, 25732–25743; J. Phys. Chem. C 2020, 124, 22392–22398] While the initial "hot" carriers indeed exhibit the light effective mass of the band edge ($m^* \approx 0.08\ m_0$) and high carrier mobility, the potential subsequent formation of polarons could significantly modulate the effective mass and thus carrier mobility. Besides lowering carrier mobility, these polarons can be energetically stabilized by lattice deformations, which suppress the recombination probability per encounter. [Rev. Mod. Phys. 2011, 83, 543; Science 2014, 343, 512–516] We do realize that such a scenario is speculative, which calls for future studies to fully explain it. We note that the formation of a polaron in GNRs and its impact on IR absorption and microwave conductivity have been recently reported. [ACS Nano 2023, 17, 18240−18252; ACS Nano 2025, 19, 25732–25743].

Action: In the revised manuscript (Lines 381-385, Page 15), we have explicitly clarified the potential role of "polaron" formation in dictating the slow exciton re-formation following the HED process as "*We note that, in the HED process, the initial non-equilibrium charge carrier mobility can be very high. However, the quasi-equilibrium mobility after ~ps carrier relaxation may be dominated by polaron formation, which significantly reduces carrier mobility. The formation of polarons has been experimentally demonstrated in GNRs*[65,66] *and may facilitate the long-lived charge-separated states observed in our longest ribbons.*"

• Related to this, the Bjerrum-length concept has been reconsidered. In the current discussion, important points relevant to a low-dimensional material are not discussed. This includes

• role of dimensionality in modifying the coulomb interaction
• local and anisotropic dielectric screening near the GNR
• the fact that carriers are initially 'hot', whereas R_B is defined using k_BT.

Response: We sincerely thank the reviewer for this critical comment regarding the dimensionality of electrostatic interactions. We believe that the essence of this comment is closely related to Comment 2 by Reviewer 1. We fully agree that, in a strictly one-dimensional electrostatic problem, the interaction law differs qualitatively from the standard $1/r$ Coulomb form. For a perfect 1D system with electrostatic confinement (e.g., in vacuum), the Coulomb potential scales linearly with the distance. In this sense, applying the bulk 3D Bjerrum length (R_B) to the current 1D system would indeed be an approximation (which we argue is a reasonably good one; see the discussion below). This issue is well recognized in reduced-dimensional materials, where non-local and environment-dependent screening substantially modify the interaction potential and exciton binding energies. [Phys. Rev. Lett. 2014, 113, 076802; Phys. Rev. B 2024, 109, 165425; Phys. Rev. Lett. 2016, 116, 056401]

Theoretical and experimental studies of low-dimensional nanostructures show that when the embedding medium has a higher or comparable dielectric constant, environmental screening effectively restores Coulombic ($1/r$) behavior at long range.

[JETP Lett. 1979, 29, 658−660; Phys. Rev. Lett. 2014, 113, 076802; Nat. Commun. 2017, 8, 15251] In particular for 1D GNR systems, a crossover from short-range to long-range screening behavior is central to determining the relevant length scale, as indicated by the work of Rocha et al. [Phys. Rev. B 2024, 109, 165425]: the authors showed that the Coulomb interaction in quasi-1D GNR systems embedded in a dielectric medium exhibits two regimes. At short distances (e.g., comparable to the ribbon width ~1.7 nm in our case and/or the estimated exciton size of ~ 1 nm from our previous work [Nano Lett. 2020, 20, 2993], the interaction is strongly enhanced and expected to approach a quasi-1D linear form. This short-range enhancement accounts for the large exciton binding energy we observed (600 ~ 700 meV). At large separations (>> ribbon width, e.g., R_B estimated in our case), the interaction is effectively screened by the bulk dielectric constant of toluene to follow the characteristic $1/r$ scaling. We hope these discussions address the first three comments by the reviewer.

For the last one related to “the fact that carriers are initially 'hot', whereas R_B is defined using k_BT.”, in our hot exciton dissociation (HED) mechanism, the nascent electron-hole pair is indeed initially "hot" with sufficient kinetic energy to overcome the short-range, strong (quasi-)1D potential. The decisive criterion for efficient charge separation over long distances with long-lived nature, however, is whether the pair can separate, before cooling down, to a distance where the remaining Coulomb attraction (now solvent-screened) falls below k_BT. As this critical length scale is much longer than the exciton length scale, it is physically consistent to use the standard 3D expression ($R_B = e^2/(4\pi\varepsilon_0\varepsilon_r k_BT)$) as discussed above. This value R_B should be interpreted as the projected separation distance along the 1D ribbon axis at which the 3D-screened Coulomb potential equals the thermal energy k_BT. In this sense, both short and long-range potential play a decisive role in the “sequential” separation of charge carriers. We have clearly included these new insights into the revised manuscript.

To sum up, we argue that while short-range exciton physics is governed by the quasi-

1D nature of the ribbon, the long-range criterion for the free-carrier escaping probability is set by the 3D solvent environment. The agreement between the experimentally observed threshold (between 11 and 58 nm) and estimated $2R_B$ supports this kinetic escape model. We have clarified this point in the revised manuscript, emphasizing that R_B serves as an environment-controlled macroscopic scale rather than a dissociation criterion derived from an ideal 1D electrostatic model.

Action: In the revised manuscript, we have explicitly clarified the applicability of 3D Coulomb interactions for estimating R_B in our experimental geometry, see Lines 299-311, Page 13.

"…*Instead, we attribute the length-dependent generation of free carriers in GNRs to a kinetic process governed by the interplay between ribbon length and the effective spatial range of Coulomb attraction. The Bjerrum length* $R_B = e^2/(4\pi\varepsilon_0\varepsilon_r k_B T)$,[56–58] *reflects the distance where thermal energy matches the Coulomb potential between carriers, where* ε_r, k_B, *and* T *represent the relative permittivity of the solvent, the Boltzmann constant, and the temperature, respectively. We note that charge carriers in GNRs are confined to a 1D geometry, resulting in strong excitonic effects.*[11] *As such, in the early time scale following optical excitation, excitation above the bandgap generates transient "hot" free carriers, and only those with sufficiently high kinetic energy can effectively overcome the strong short-range 1D Coulomb potential for charge separation: greater photon energies yield excess kinetic energy, allowing electrons and holes to separate more effectively. Following this "initial" dissociation process, however, the decisive criterion for generating long-lived free carriers is then whether they can separate further, before cooling, to a distance where the remaining, solvent-screened Coulomb attraction drops below* $k_B T$. *As this critical length scale is much longer than the exciton length scale (on the order of 1 nm), the long-range interaction is effectively mediated by the surrounding 3D liquid environment. Consequently, we consider a fully solvent-screened 3D Coulomb potential, rather than a strictly 1D form, for estimating* R_B."

Minor points:

• In Fig. 2d: only very few points with different pump energies are measured for the 6 nm GNRs. Why does this differ from the other GNRs and what why is the error bar (not visible for 6 nm GNRs) so much smaller? Scaling factors should be simplified by choosing one (unscaled) reference curve. Also, mixing multiplication and division factors adds unnecessary confusion.

Response and Action: We thank the reviewer for pointing out the issue with Figure 2d.

The 6-nm GNR dataset in Figure 2d contains fewer pump-energy points because, for this shortest ribbon, the THz photoconductivity amplitude is substantially weaker, and the signal-to-noise ratio becomes the limiting factor when scanning many excitation energies. We therefore prioritized acquiring high-quality traces at a smaller number of representative pump energies, rather than collecting a dense energy series with large statistical uncertainty. For the longer GNRs, the stronger response enabled us to sample a wider range of pump energies while maintaining comparable data quality. In the originally submitted version, the error bars for the 6 nm GNR data were omitted from the plot. This has been corrected in the revised Figure 2d, where the uncertainties for all datasets are now explicitly shown. The error bars represent the standard error of the maximum value extracted from multiple measurements.

Finally, the scaling factors in Figure 2d are introduced solely to facilitate visual comparison. Specifically, the maximum value of the real part of the THz photoconductivity for each AHM-GNR length is rescaled to unity so that the excitation-energy dependence and threshold behavior for all AHM-GNRs can be directly compared. Multiplying the data by the indicated factors restores the absolute amplitudes, which are consistent with the unscaled data shown in Figure 2a (excited at 3.1 eV as an example).

• In Fig 4d: Solid lines are described as single exponential functions. This needs further details. What exact function is used to fit the delay time-dependent exciton fraction?

Response: The solid lines in Figure 3b and Figure 4d correspond to a single-exponential rise function used to describe the temporal build-up of the exciton fraction. Specifically, the data are fitted using $y(t) = A\cdot(1 - e^{-t/\tau})$, where A is the saturation amplitude and τ is the characteristic rise time. This functional form follows from a minimal two-state rate model. In this picture, photoexcitation initially populates free-carrier states, which subsequently convert to excitonic states at approximately constant rate k. The corresponding rate equations are $dn_F / dt = -k\cdot n_{EX}$, $dn_{EX} / dt = k\cdot n_F$, with the initial conditions $n_F(0) = n_0$ and $n_{EX}(0) = 0$. Solving these equations gives $n_{EX}(t) = n_0\cdot(1 - e^{-kt})$, so that the exciton fraction rises exponentially toward saturation. The form used in the manuscript is the generalized expression $y(t) = A\cdot(1 - e^{-kt})$.

Physically, this description represents an effective picture in which the dominant dynamics within the experimental time window are governed by a single characteristic conversion process. The appearance of a clean single-exponential rise indicates that additional channels (such as back-conversion, recombination, or multiple intermediate states) do not introduce distinct competing time scales under the present conditions. We have added the explicit fitting function and this physical interpretation to the manuscript to clarify the origin and meaning of the exponential behavior.

Action: Following the reviewer's suggestion, we have now updated the figure captions for Figure 3b and Figure 4d in the revised manuscript, see Line 288, Page 12, and Line 386, Page 16.

"*Figure 3. …(b) The relative percentage of excitons as a function of the pump-probe delay time. The error bars are calculated via the standard error propagation from the uncertainties of A(t) and B(t). The solid lines represent fits to a single-exponential rise function,* $y(t) = A(1 - e^{-kt})$, *where A is the saturation amplitude and k is the characteristic rise rate. This functional form describes the temporal build-up of the*

exciton fraction, reflecting a single characteristic conversion process from free carriers to excitonic states.

…

Figure 4. … (d) The relative percentage of excitons as a function of the pump-probe delay time for three GNRs. The error bars are calculated via the standard error propagation from the uncertainties of $A(t)$ and $B(t)$. The solid lines represent fits to a single exponential rise function ($y(t) = A(1 - e^{-kt})$)."

Reviewer #3 (Remarks to the Author):

"The manuscript presents a clear storyline, and most of the claims are well supported by theory. The authors investigate a strong ~1 eV tightly bound exciton in ~1 nm-wide GNRs, which enables hot exciton dissociation (HED) to remain stable in longer ribbons (lengths > 2 × 20 nm) compared to other low-dimensional organic material systems where the exciton binding energy (E_B) is typically below 1 eV.
They perform ultrafast terahertz (THz) conductivity measurements on:
• 6, 11, and 58 nm AHM-GNRs with ~0.08 m_0 carrier effective mass,
• 70 nm CMGNR with ~0.8 m_0 effective mass,
• 43 nm HTGNR with similar effective mass to AHM-GNR.
These measurements systematically demonstrate that for GNRs with high mobility (i.e., low effective mass), HED can exist and contribute up to ~30% of the total carrier in the ribbon.
The study of hot excitons using THz photoconductivity, decomposed into free-carrier and excitonic contributions via a Drude–Smith plus low-frequency excitonic model, and the extraction of exciton fractions versus time for different lengths is intuitive and well executed.
This work extends the previous story of "strong exciton" in GNRs (Nano Lett. 20, 2993–3002 (2020) from the same group) from static binding energy and for different GNR formation to length-dependent hot carrier dissociation. The experimentally grounded Bjerrum-length framework in a strongly excitonic 1D system is also valuable. This is likely to interest not only the GNR community but also researchers in broader excitonic material platforms. Most of the claims and conclusions appear justified"
and …
"Overall Assessment
The methodology is clearly described, the measurements appear sound, and the work meets the standards of the field. The provided methods and references should enable experimental reproducibility."

Response: We thank the reviewer for the positive assessment of our manuscript and for the useful and constructive comments.

Questions for the Authors

1. Width variations and localization

Given that the width of the GNRs can vary between 1 and 1.7 nm, could atomic-scale variations localize excitons? These fluctuations could behave as 1D quantum wells. If so:

♣ Could they contribute to the reactive negative imaginary conductance?

♣ How can their contribution be distinguished from that of free excitons?

Response: We thank the reviewer for this question. The first comment is related to whether the atomic-scale variation in the width direction can result in short-range potential fluctuations so that photoexcitation leads to exciton localization to the nanometer-scale segments. To investigate this mechanism further quantitatively, we extracted the microscopic exciton polarizability (α) of AHM-GNR (11 nm) directly from our excitonic-dominated frequency-dependent complex photoconductivity spectra, as shown in the revised Supporting Information Figure S6. Physically, the bound excitons manifest as a purely polarizable species, contributing primarily to the imaginary part of the conductivity, which is proportional to the dielectric change. By modeling the photoexcited GNR dispersion as a dielectric composite and applying the Clausius-Mossotti relation [Phys. Rev. Lett. 2004, 92, 196601; Phys. Rev. B 2005, 71, 125201; Nat. Mater. 2006, 5, 861−864], we determined an intrinsic exciton polarizability of ~6 nm^3. This exceptionally large value provides a decisive length-scale comparison: since polarizability scales with the wavefunction volume ($\alpha \approx 4\pi r^3/3$), a value of ~6 nm^3 implies a coherence length ($r \approx 1$ nm) that is significantly larger than the scale of local atomic fluctuations (< 0.7 nm) along the ribbon. This result provides a rough estimation of the exciton localization length scale and indicates that the exciton wavefunction is spatially extended enough to “average over” the short-range fluctuation.

Second, the reviewer wondered, “Could they contribute to the reactive negative imaginary conductance?”. As discussed in the manuscript, thanks to the large E_B and small THz frequency spectrum window in our study, a negative imaginary photoconductivity is the expected signature of bound electron-hole pairs that are

polarized non-resonantly by the THz field, independent of the "spatial" localization (and thus mobility of the exciton) or delocalization features commented by the reviewer.

Last but not least, the reviewer commented "How can their contribution be distinguished from that of free excitons". Due to the charge-neutral nature of excitons (in particular with a large binding energy), they do not absorb any THz radiation in the low-frequency window for both localized and free excitons; as such, our study provides no direct evidence on the exciton transport scale. To provide further evidence on the exciton localization/delocalization, a direct characterization of spatial-temporal mapping of exciton diffusion with a sub-ps time scale is needed in a single GNR domain: this is extremely challenging and is also out of the focus of the current work.

Action: In the revised manuscript (Lines 341-345, Page 14), we have briefly clarified that the exciton wavefunction extends beyond the scale of local width fluctuations. To quantitatively support this, the detailed extraction of the exciton polarizability and coherence length has been added to the revised Supporting Information, see Figure S6, Page S7.

"*...Notably, R_B extends far beyond conventional quantum confinement (represented by the Bohr radius of roughly 1 nm), offering a more accurate framework for understanding charge separation in GNRs. In contrast, the equilibrium exciton size remains much smaller, at approximately 0.7 nm, due to the localized nature of excitons in these systems.*[11] *Despite this strongly bound nature, we estimate the exciton spatial extent (polarizable length) to be ~1.1 nm, derived from the exciton polarizability (see Figure S6). This extension exceeds the local atomic-scale width fluctuations (< 0.7 nm). As a result, the exciton wavefunction effectively averages over these short-range variations, preventing strong exciton localization by sub-nanometer geometric defects.*"

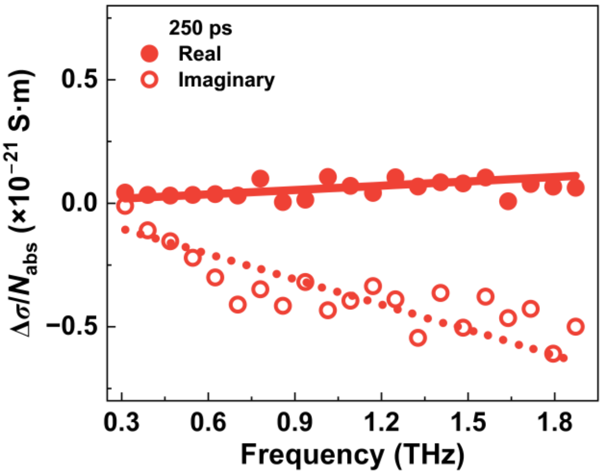

Figure S6. Extraction of the polarizability for the 11 nm AHM-GNR. Frequency-dependent complex THz photoconductivity measured at ~250 ps after the maximum photoconductivity. Solid and open circles represent the experimental real and imaginary parts of the photoconductivity, respectively. At this delay time, the frequency dispersion is dominated by bound excitons, which appear as purely polarizable species with a near-zero real component and a negative imaginary component. The solid and dashed lines represent the modeled real and imaginary conductivities, respectively. These theoretical curves are generated by modeling the GNR dispersion as a dielectric composite using the Clausius-Mossotti relation.[8–10] By matching the theoretical conductivity to the experimental data (specifically the linear frequency dependence of the imaginary part), an average exciton polarizability $\alpha \approx 6\ \text{nm}^3$ is extracted, implying an exciton spatial extension of ~1.1 nm.

2. Applicability to device platforms

Since the suspension GNR system lacks a substrate and electrical contacts, how does this translate to practical applications such as photodetectors, solar cells, and phototransistors? These devices must be substrate-supported and electrically (or capacitively) contacted, and these factors could significantly modify mobility, screening, and heat dissipation.

Response: The present study is not intended to replicate full device architecture, but rather to extract design-relevant physical insights under well-controlled conditions. By studying suspended, contact-free GNRs, we are able to isolate the intrinsic non-equilibrium processes that govern hot-carrier transport and exciton dissociation, without the additional complexity introduced by substrates and contacts.

From a device-design perspective, the key outcome of this work is the identification of ribbon length as a critical intrinsic parameter that controls whether photoexcited carriers

can dissociate and remain mobile on ultrafast time scales. Our results show that once the ribbon length exceeds a characteristic scale, long-lived mobile carriers can be generated before recombination or localization dominates. This establishes a fundamental constraint that should be satisfied with the specific device platform.

In practical photodetectors, solar cells, or phototransistors, substrates and electrical contacts will inevitably modify the absolute mobility, screening, and heat dissipation through additional scattering channels and substrate dielectric effects. However, these extrinsic factors act on top of the intrinsic carrier dynamics identified here. In this sense, the suspended measurements provide an upper bound for free carrier generation in GNRs and a reference point for what can be achieved when extrinsic losses are minimized. The observed length dependence, therefore, offers a concrete guideline for device design: when GNRs are sufficiently long, they enable efficient hot-carrier dissociation prior to contact-induced or substrate-induced relaxation.

Action: We have clarified this perspective in the revised manuscript to emphasize the relevance of our findings for guiding realistic device applications, see Lines 413-418, Page 17.

"*...Finally, the ability to efficiently generate long-lived free carriers in GNRs, as revealed by the critical dependence on ribbon length and band dispersion, is directly relevant for their implementation in optoelectronic devices such as photodetectors, solar cells, and phototransistors. While practical solid-state architectures inevitably introduce substrates and electrical contacts that modify absolute mobility and dielectric screening, our contact-free measurements isolate the intrinsic non-equilibrium processes governing charge separation. These suspended measurements effectively provide a fundamental reference point for performance when extrinsic losses are minimized. By exceeding the R_B threshold and optimizing m^*, GNRs can sustain free-carrier populations even in strongly excitonic regimes, suggesting strategies to enhance photocurrent and quantum efficiency in real nanoribbon-based device architectures.*

These insights provide actionable guidance for selecting or engineering GNRs for integration into solid-state device platforms, where overcoming rapid exciton recombination is pivotal for efficient operation."

3. Regarding Fig. S1

Why is the photoconductivity at 0.5 ps for the 11 nm AHM-GNR approximately twice lower than that for the 6 nm and 58 nm samples?

Response and Action: The photoconductivity amplitudes at 0.5 ps in the originally shown traces are not directly comparable across the three samples because they were plotted without normalization to the number of absorbed photons density (N_{abs}). In the revised version, we normalize photoconductivity by the N_{abs} for all three samples. As seen in revised Supporting Information Figure S3, the photoconductivity magnitude shows a clear length-dependent trend: the THz photoconductivity increases with increasing AHM-GNR length for a fixed absorbed photon density. This normalization, along with the corresponding revised figure, has been added to Supporting Information Figure S3 to enable a meaningful comparison of the intrinsic response.

Suggestions
• A TEM image of the GNRs would be a strong experimental confirmation of uniformity for the synthesized structures.

Response: We appreciate the constructive suggestion. Because AHM-GNR possesses a width less than 2 nm, they are difficult to identify on copper grids or ultra-thin carbon films for TEM observation. Actually, the uniformity of these AHM-GNR samples was already carefully confirmed in our previous study [J. Am. Chem. Soc. 2018, 140, 10416; Nano Lett. 2020, 20, 5, 2993; Nano Lett. 2024, 24, 797]. The raw AHM-PP samples were synthesized in our previous work and further purified by recycling gel permeation chromatography (recycling GPC) to obtain three AHM-PP samples with narrow molecular weight distribution (*Đ*), as shown in the revised Supporting Information Table S1. The length of AHM-GNR was determined by the number-average molecular weight (M_n) of AHM-PP and the molecular weight of the polymeric monomer. Second, the AFM phase image of self-assembled domains of the AHM-GNRs on HOPG confirmed the good width uniformity of AHM-GNRs. Moreover, the low-frequency region includes a characteristic peak at ~258 cm^{-1} in Raman spectra, associated with the radial breathing-like layer mode, further confirming the narrow (< 2 nm, near to the theoretical width of the GNR backbone) and atomically precise structure of AHM-GNRs [J. Am. Chem. Soc. 2018, 140, 10416]. In a word, the AHM-GNRs samples possess good width and length uniformity.

Action: We have added the following sample information in the revised manuscript (see Lines 142-144, Page 6, and Lines 149-151, Page 6) and Supporting Information (see Page 10).

"*...In this work, the studied GNRs have a fixed width of 1.7 nm and average lengths of 6, 11, and 58 nm controlled by the number of repeating units as 4, 8, and 42, respectively. The corresponding molecular weights and polydispersities of the polymer precursors are summarized in Table S1.*"

"*...The three available ribbon lengths enable us to investigate length-dependent charge-carrier dynamics in AHM-GNRs spanning aspect ratios by an order of magnitude (from ~ 3 to 35). The uniformity of AHM-GNR was demonstrated by atomic force microscopy and Raman spectroscopy in our previous study.*[42]"

Table S1. Sample information of three AHM-GNR with different lengths:molecular weight M_n, polydispersity *Đ* and length.

Sample name		M_n (g/mol)	*Đ*	Length (nm)
AHM-GNR-1	AHM-PP-1	13500	1.40	~6
AHM-GNR-2	AHM-PP-2	23100	1.20	~11
AHM-GNR-3	AHM-PP-3	124000	1.50	~58

The number-average molecular weights of the AHM-PP samples were determined by gel permeation chromatography (GPC) using a Wyatt Optilab DSP differential refractometer with tetrahydrofuran (THF) as the eluent and standard polystyrene as the reference.

• Future studies (not needed for this paper) could investigate substrate effects on hot exciton dissociation, since mobility, charge screening, and heat dissipation would directly impact exciton stability (possibly reducing the stability) and therefore device performance. Understanding this dependence could lead to device configurations that are better match for photodetector, solar cell, and phototransistor applications.

Response: We thank the reviewer for this constructive suggestion. We fully agree with the reviewer that moving forward from our present solution-phase measurements to supported or encapsulated GNR films will introduce additional control parameters, such as dielectric screening, interfacial disorder/doping, and heat dissipation pathways, inter-GNR coupling, which can strongly influence exciton dynamics and the efficiency of hot-exciton dissociation. Environmental screening is known to renormalize Coulomb interactions in low-dimensional systems and can substantially modify exciton binding and charge-separation energetics. [Nat. Commun. 2017, 8, 15251; Phys. Rev. Lett. 2014, 113, 076802; Phys. Rev. B 2024, 109, 165425; Phys. Rev. Lett. 2016, 116, 056401] In addition, substrate coupling can alter cooling and relaxation dynamics, which directly impact whether initially hot populations can access charge-separated configurations

before relaxing. [Phys. Rev. B 2012, 86, 045413; Rev. Mod. Phys. 2011, 83, 543.]

We also agree with the reviewer that these substrate/interface effects are outside the scope of the present manuscript, which focuses on intrinsic free-carrier generation and recombination of dispersed GNRs in dielectric environment. We hope that our work motivates further investigation of the free carrier generation dynamics in GNRs supported by substrates, directly relevant for their optoelectronic applications.

Action: We have added a brief outlook statement noting that systematic studies on supported/encapsulated geometries (varying substrate permittivity and thermal coupling) would be a natural next step toward device-relevant configurations for photodetectors, solar cells, and phototransistors, see Lines 423-436, Page 18.

"*While the present study establishes the intrinsic physical limits of free-carrier generation in isolated GNRs, transitioning these nanomaterials into practical solid-state architectures necessitates understanding their interaction with the immediate environment. Moving to supported or encapsulated GNR films introduces additional control parameters, including dielectric screening, interfacial disorder, heat-dissipation pathways, and inter-GNR coupling. Environmental screening is known to renormalize Coulomb interactions in low-dimensional systems, thereby substantially modifying exciton binding and charge-separation energetics.*[67] *Furthermore, substrate coupling can alter cooling and relaxation dynamics, directly dictating whether hot populations can successfully access charge-separated configurations before relaxing to strongly bound states. Therefore, systematic studies of these supported geometries (specifically varying the substrate permittivity and thermal coupling) represent a natural and critical next step toward device integration of GNRs. Such efforts will bridge the gap between intrinsic photophysics and the rational design of optimal device configurations for next-generation photodetectors, solar cells, and phototransistors.*"

Point-by-point response to referees' comments

Manuscript ID: NCOMMS-25-098713A
Manuscript Type: Article
Title: Hot Exciton Dissociation in Graphene Nanoribbons
Author(s): Guanzhao Wen, Fugui Xu, Alexander Tries, Wenhao Zheng, Lucia Di Virgilio, Shuai Fu, Xinyu Chen, Lin Yang, Zijie Xiao, Mathias Kläui, Silvio Osella, Ji Ma, Xu Wang, Xinliang Feng, Yiyong Mai, Mischa Bonn, and Hai I. Wang.

REVIEWER COMMENTS

Reviewer #1 (Remarks to the Author):

"The authors have provided thorough responses, offering reasonable arguments as well as complementary experimental measurements (where appropriate) to address the points raised by the three reviewers. In particular, I am satisfied with how the authors have addressed my main concern regarding the Bjerrum length approximation. In the revised manuscript, the validity of this approximation is now clearly stated.
Although the inclusion of Ref. 11 is essential to support their response, I believe that the key statement presented in the replies to Referee #1 and Referee #2 should also be incorporated into the revised manuscript, i.e.,
"In our system, the GNRs are narrow but embedded in a liquid solvent with a comparable or higher dielectric constant. Under these conditions, the short-range excitonic physics is governed by the quasi-1D, strongly bound regime, whereas at larger separations the Coulomb interaction is expected to be dominated by solvent screening and can be reasonably described by a 3D-screened potential [Phys. Rev. B 2024, 109, 165425]."

Including this statement would further strengthen the manuscript. With this minor revision, I consider the paper suitable for publication in Nature Communications.

Response: We thank the reviewer for the positive assessment of our revised manuscript. We have incorporated the suggested statement into the main text to clarify the validity of using a solvent-screened 3D Coulomb potential for estimating R_B, see Lines 303-308, Page 11.

"*...As such, in the early time scale following optical excitation, excitation above the bandgap generates transient hot free carriers, and only those with sufficiently high kinetic energy can effectively overcome the strong short-range 1D Coulomb potential*

for charge separation: greater photon energies yield excess kinetic energy, allowing electrons and holes to separate more effectively. Following this initial dissociation process, however, the decisive criterion for generating long-lived free carriers is then whether they can separate further, before cooling, to a distance where the remaining, solvent-screened Coulomb attraction drops below k_BT. Moreover, the GNRs are narrow but embedded in a liquid solvent with a comparable or higher dielectric constant. Under these conditions, the short-range excitonic physics is governed by the quasi-1D, strongly bound regime, whereas at larger separations the Coulomb interaction is expected to be dominated by solvent screening and can be reasonably described by a 3D-screened potential.[59] *Consequently, we estimate R_B using the solvent-screened 3D Coulomb interaction rather than a strictly 1D potential.*”

Reviewer #3 (Remarks to the Author):

The authors have addressed the concerns and suggestions I raised in the previous round of review. The revised manuscript is now clear, with well-supported physical interpretations and improved clarity regarding the practical applications. I have no further questions and recommend this manuscript for publication.

Response: We sincerely thank the reviewer for the positive assessment of our revised manuscript and for recognizing the improvements made in the revision. We are grateful for the recommendation for publication.
